# Mitochondrial (mt)DNA–cyclic GMP–AMP synthase (cGAS)–stimulator of interferon genes (STING) signaling promotes pyroptosis of macrophages via interferon regulatory factor (IRF)7/IRF3 activation to aggravate lung injury during severe acute pancreatitis

**DOI:** 10.1186/s11658-024-00575-9

**Published:** 2024-04-27

**Authors:** Yiqiu Peng, Yuxi Yang, Yingying Li, Tingjuan Shi, Ning Xu, Ruixia Liu, Yingyi Luan, Yongming Yao, Chenghong Yin

**Affiliations:** 1 Department of Central Laboratory, Beijing Obstetrics and Gynecology Hospital, Capital Medical University, Beijing Maternal and Child Health Care Hospital, No. 251 Yaojiayuan Road, Chaoyang District, Beijing, 100026 China; 2 Translational Medicine Research Center, Medical Innovation Research Division and Fourth Medical Center of the Chinese People’s Liberation Army (PLA) General Hospital, Beijing, 100048 China

**Keywords:** Severe acute pancreatitis, Macrophage, NLRP3, Pyroptosis, IRF7, IRF3

## Abstract

**Background:**

Macrophage proinflammatory activation contributes to the pathology of severe acute pancreatitis (SAP) and, simultaneously, macrophage functional changes, and increased pyroptosis/necrosis can further exacerbate the cellular immune suppression during the process of SAP, where cyclic GMP–AMP synthase (cGAS)–stimulator of interferon genes (STING) plays an important role. However, the function and mechanism of cGAS–STING in SAP-induced lung injury (LI) remains unknown.

**Methods:**

Lipopolysaccharide (LPS) was combined with caerulein-induced SAP in wild type, *cGAS *^*−/−*^ and *sting *^*−/−*^ mice*.* Primary macrophages were extracted via bronchoalveolar lavage and peritoneal lavage. Ana-1 cells were pretreated with LPS and stimulated with nigericin sodium salt to induce pyroptosis in vitro.

**Results:**

SAP triggered NOD-, LRR-, and pyrin domain-containing protein 3 (NLRP3) inflammasome activation-mediated pyroptosis of alveolar and peritoneal macrophages in mouse model. Knockout of *cGAS*/*STING* could ameliorate NLRP3 activation and macrophage pyroptosis. In addition, mitochondrial (mt)DNA released from damaged mitochondria further induced macrophage STING activation in a cGAS- and dose-dependent manner. Upregulated STING signal can promote NLRP3 inflammasome-mediated macrophage pyroptosis and increase serum interleukin (IL)-6, IL-1β, and tumor necrosis factor (TNF)-α levels and, thus, exacerbate SAP-associated LI (SAP-ALI). Downstream molecules of STING, IRF7, and IRF3 connect the mtDNA–cGAS–STING axis and the NLRP3–pyroptosis axis.

**Conclusions:**

Negative regulation of any molecule in the mtDNA–cGAS–STING–IRF7/IRF3 pathway can affect the activation of NLRP3 inflammasomes, thereby reducing macrophage pyroptosis and improving SAP-ALI in mouse model.

**Supplementary Information:**

The online version contains supplementary material available at 10.1186/s11658-024-00575-9.

## Introduction

Acute pancreatitis (AP) is one of the most common acute abdominal diseases of the digestive system, with a complex etiology and prone to relapse [1]. Patients with severe disease are susceptible to multiple organ insufficiency and even sepsis, which is associated with poor prognosis. Currently, there is no specific drug for clinical treatment, which is mainly based on fluid resuscitation, nutritional support, organ function protection, anti-infection, and other symptomatic treatment [2]. Therefore, exploring the pathological mechanisms with potential therapeutic value is crucial for developing scientific disease management strategies and reducing the disease burden.

Macrophages are critical in systemic inflammation and organ injury in severe acute pancreatitis (SAP), differentiating into various phenotypes for diverse functions [3, 4]. These cells, the predominant immune responders in AP, migrate towards the pancreas, with their infiltration more significantly correlating with pancreatic injury and necrosis than neutrophil infiltration [5]. In AP, macrophages are activated in different tissues, contributing to the immune response by secreting cytokines and extracellular vesicles. The dynamic changes in surface and functional markers of the pancreatic CD206^+^ macrophage populations are associated with disease severity and recovery. These monocyte/macrophage subsets, exhibiting distinct phenotypic and functional characteristics, are potential biomarkers for disease monitoring [6]. Modulating macrophage polarization may protect against pancreatic injury in SAP [7, 8]. Emodin has been shown to reduce alveolar macrophage activation, thereby ameliorating SAP-associated lung injury (SAP-ALI) [9]. Consequently, managing macrophage behavior could be an effective therapeutic strategy for SAP.

Pyroptosis, a form of inflammation-related programmed cell death, was initially identified as a macrophage death pathway involving the caspase family and has since been observed in various cell types, playing a significant role in inflammation, tumors, and other diseases [10]. Studies have shown that pyroptosis contributes to the development of AP [11], and some drugs have protective effects against AP by inhibiting pyroptosis-related signaling pathways [12, 13]. The NOD-, LRR, and pyrin domain-containing protein 3 (NLRP3) is an important cytoplasmic pattern recognition receptor that responds to multiple stimuli from the pathogen-associated molecular patterns (PAMPs) and the damage-associated molecular patterns (DAMPs). Inflammasomes, composed of apoptosis-associated speck-like protein containing a CARD (ASC) and procaspase-1, are formed, with NLRP3 inflammasome activation leading to gasdermin D cleavage by caspases [14], triggering cell death and subsequent pathophysiological responses. DNA damage, a crucial inflammation and cell damage inducer, activates the cGAS–STING signaling pathway, contributing to AP’s inflammatory damage [15]. In AP’s early stages, damaged acinar cells release DNA, activating macrophages’ cGAS–STING signaling pathway, thus initiating local inflammation. As AP progresses, peritoneal macrophage activation exacerbates the spread of local inflammation [16], with inflammatory mediators activating resident macrophages in various organs, leading to organ damage in SAP [17, 18]. NLRP3 inflammasome-mediated pyroptosis can occur through the cGAS–STING signaling pathway, associated with mtDNA leakage [19–21]. The STING signal plays a critical role in SAP progression, although the exact relationship between STING and NLRP3 remains unclear. This study aims to investigate the cGAS–STING signaling pathway’s role in regulating macrophage pyroptosis and mitigating tissue damage in SAP and to clarify the interaction between the mtDNA–cGAS–STING axis and the NLRP3–pyroptosis axis.

## Methods and materials

### Animal model of SAP

**Fig. 1:**
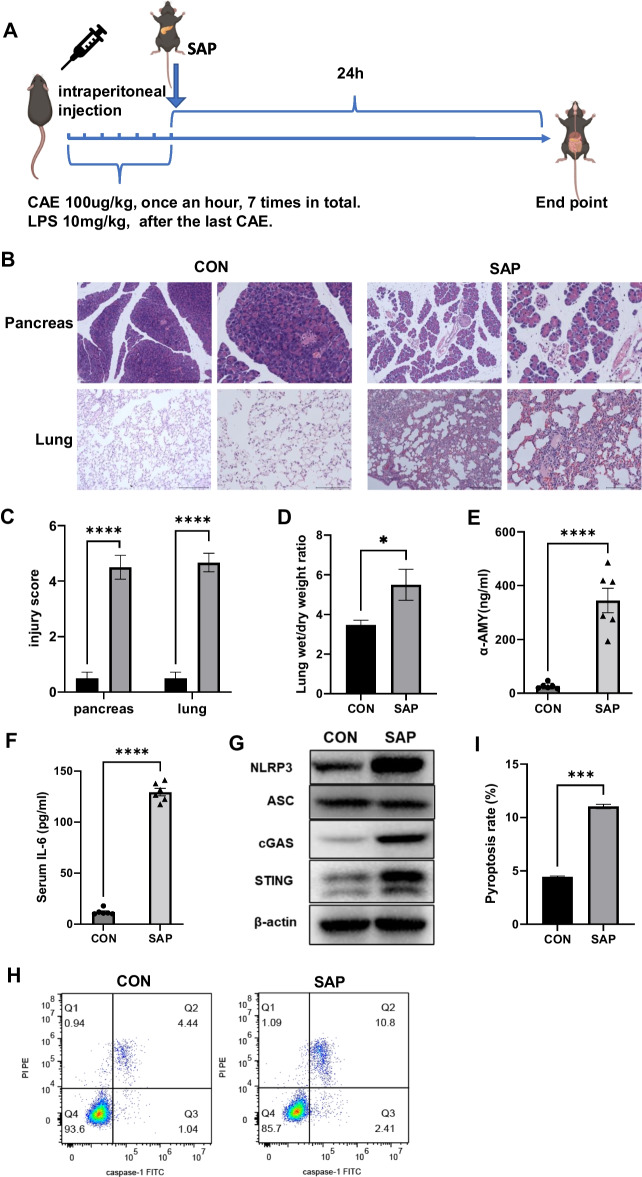
cGAS–STING signaling activation accompanied by increased alveolar macrophage pyroptosis. **A** Flow chart for constructing the animal model of SAP (*n* = 6 per group). **B** Hematoxylin and eosin staining was used to observe the pancreatic and lung injury. **C** Injury score of pancreas and lungs. **D** The wet/dry weight ratio of the lungs. **E** Serum amylase level. **F** Serum IL-6 level. **G** WB of NLRP3, ASC, cGAS, and STING. **H** Flow cytometry was used to detect the pyroptosis of alveolar macrophages. **I** Pyroptosis rate of alveolar macrophages. *p < 0.05, **p < 0.01, ***p < 0.001, ****p < 0.0001

cGAS-KO (*cGAS*^*−/−*^) mice and Sting1-KO (*sting*^*−/−*^) mice were purchased from GemPharmatech (Nanjing, China). Wild type C57BL/6J mice were obtained from SPF (Beijing) BIOTECHNOLOGY Co., Ltd. All mice were acclimatized for 7 days before induction initiation in the models. SAP was induced by intraperitoneal injection of caerulein (CAE, 100 µg/kg, once an hour, seven times in total; AbMole, M9316) and lipopolysaccharide (LPS, 10 mg/kg following the last injection of CAE; Sigma-Aldrich, L2630) (Fig. 1A). Serum, primary macrophages, and pancreatic and lung tissues were collected 24 h after modeling.

### Cell culture

Primary alveolar macrophages were extracted via bronchoalveolar lavage, as previously described [22]. Primary peritoneal macrophages were extracted via peritoneal lavage 24 h after modeling, which was performed with Dulbecco’s modified Eagle’s medium containing 10% fetal bovine serum, 1 × penicillin, and 1 × streptomycin. Murine macrophage cells Ana-1 were purchased from MeisenCTCC. For in vitro experiments, Ana-1 cells were pretreated with LPS (1 mg/mL) for 4 h and then stimulated with 10 µM nigericin sodium salt (MCE, HY-100381) for 1 h to induce pyroptosis. Ana-1 cells were treated with 100 ng/mL ethidium bromide (EthBr) (MCE, HY-D0021) for 72 h to cause mtDNA depletion. The grouping was performed as follows: for the normal control (CON), only solvent was added; for L+N, pyroptosis was induced by the LPS + nigericin sodium salt as described above; for EthBr, EthBr was applied to induce mitochondrial depletion in cells using the protocol described above; and for EthBr + L+N, mitochondria were depleted by EthBr followed by L+N induction of pyroptosis.

### RNA-sequencing and bioinformatics analysis

Blood was collected from control (CON) mice and SAP mice, and high-throughput sequencing was performed by Novogene Co., Ltd. (Beijing, China). TRIzol reagent (Invitrogen) was used for total RNA extraction. The mRNA library was established using NEBNext^®^ Ultra™ RNA Library Prep Kit for Illumina^®^ (NEB, USA, E7530L), followed by sequencing using the Illumina NovaSeq 6000. The data measured using the high-throughput sequencer were converted into sequence data (reads) with CASAVA base recognition. Fragments per kilobase per million was used to standardize the read counts for each gene calculated with featureCounts (v1.5.0-p3). Differential expression analysis between the two groups was conducted using the DESeq2 R package (v1.20.0), setting an adjusted *p*-value < 0.05 and |logFC|> 1.0 as criteria for significant differential expression. The clusterProfiler R package (v3.8.1) was utilized to assess the statistical enrichment of differentially expressed genes (DEGs) in the Kyoto Encyclopedia of Genes and Genomes (KEGG) analysis. The protein–protein interaction (PPI) network was created using the Search Tool for the Retrieval of Interacting Genes official website (https://cn.string-db.org/). All raw data have been stored in the NCBI Gene Expression Omnibus (GEO) database (GSE244335).

### Cell transfection

Ana-1 cells were plated in six-well plates until 50–60% confluence was achieved. They were transfected with siRNAs using ^jetPRIME®^ (Polyplus, 101000046) according to the manufacturer’s protocol; si-NC (RiboBio, China) was used as a negative control in vitro. Pyroptosis was induced via sequential administration of LPS and nigericin 48 h after transfection. C57BL/6 mice were administered tail vein injection of 10 nmol/20 g siRNA to knockdown the expression levels of IRF3 and IRF7 on day 3 and day 1; si-NC (RiboBio, China) was used as a negative control in vivo. The mouse model of SAP was induced via an intraperitoneal injection of LPS combined with CAE on day 0. The sequences of siRNA are listed in Additional file 1: Table S1.

We plated Ana-1 cells in six-well plates until 90% confluence was achieved. They were transfected with 10 µg/mL 2′,3′-cGAMP (MCE, HY-100564) or 5 µg/mL poly(dA:dT) (Invivogen, tlrl-patn) for 6 h using ^jetPRIME®^ (Polyplus, 101000046) to activate the STING signal.

### Histopathological analysis

Pancreatic and lung tissues were fixed in 4% paraformaldehyde for 48 h, followed by dehydration and embedding. Sections of paraffin-embedded tissues, 5-µm thick, were deparaffinized and stained with hematoxylin and eosin. Pancreatic edema, inflammation, hemorrhage, and necrosis were examined under an optical microscope, with scores from 0 to 4 points for each criterion. Pulmonary edema, alveolar congestion, infiltration of inflammatory cells, and atelectasis were similarly evaluated, assigning 0 to 4 points for each parameter. The specific scoring criteria for pancreatic and lung injury were defined as 0 for normal, 1 for mild, 2 for moderate, 3 for severe, and 4 for extremely severe. The cumulative total score for all items was subsequently calculated. A standard immunohistochemistry (IHC) protocol was used for the lung tissue. Briefly, the sample was blocked and incubated overnight with primary antibodies against IRF3 (Abcam, ab68481, 1:500) and IRF7 (Affinity, #DF7503, 1:100) at 4 °C. Then, the sections were washed thrice with phosphate-buffered saline (PBS) and incubated with a horseradish peroxidase (HRP)-conjugated secondary antibody for 60 min. Finally, immunohistochemical staining was performed with hematoxylin counterstained with diaminobenzidine. According to the staining intensity: 0 was negative, 1 was weakly positive, 2 was positive, and 3 was strongly positive. The extent of staining was scored as follows: 1 point for ≤ 25%, 2 points for 26–50%, 3 points for 51–75%, and 4 points for > 75%. The final IHC score was obtained by multiplying the intensity with the extent of staining scores.

### Flow cytometry

The FAM-FLICA^®^ Caspase assays (Immunochemistry, ICT-98) were employed to identify cell pyroptosis. Cells positive for both caspase-1 and propidium iodide (PI) were classified as typical pyrolytic cells. To detect primary mouse alveolar macrophages in bronchoalveolar lavage fluid, CD170 (Siglec F) monoclonal antibody (1RNM44N), conjugated with Alexa Fluor™ 700, eBioscience™ (Invitrogen, 56–1702–82) was used. For the identification of frame-selected mouse primary peritoneal macrophages in peritoneal lavage fluid, V450 rat anti-CD11b (BD Horizon™, cat. no. 560455) and PE/Cy7^®^ anti-F4/80 (abcam, ab218761) were utilized.

### Measurement of cytokines and amylase

Whole blood samples were centrifuged at 3000 rpm for 15 min after resting overnight at 4 °C to obtain the serum, which was frozen at −80 °C. We seeded 2 × 10^5^ Ana-1 cells in each well of a 24-well plate and collected the cell supernatant after exposure to different stimuli. Mouse IL-1β ELISA Kit (Hangzhou Lianke Biotechnology Co., Ltd. EK201B), Mouse IL-6 ELISA Kit (Hangzhou Lianke Biotechnology Co., Ltd. EK206), Mouse TNF-α ELISA Kit (Hangzhou Lianke Biotechnology Co., Ltd. EK282), and α-Amylase Assay Kit (Nanjing Jiancheng Biological Engineering Research Institute, C016-1-1) were used to measured cytokines and serum amylase according to the manufacturer’s protocol.

### Real-time quantitative polymerase chain reaction (PCR) analysis

Total RNA was extracted from Ana-1 cells using HiPure Total RNA Mini Kit (Magen, R4111–02). We used the PrimeScript ™ RT reagent Kit (TaKaRa, cat. no. RR037A) to reverse transcribe RNA samples (1 µg) into cDNA. Hieff^®^ QPCR SYBR Green Master Mix (Yeasen, 11201ES03) was used for real-time quantitative PCR. The relative gene expression level was normalized with the internal parameter (β-actin) using the comparative cycle threshold (Ct) method (2^−ΔΔCt^).

For the analysis of mtDNA copy number in the cytoplasm, we divided the cells into two equal parts; one part was used to isolate the cytoplasm [23] and the other was used to extract the total cellular DNA using the animal DNA column extraction kit (Beijing BioRab Technology Co. Ltd, BTN71206). The whole mtDNA copy number was used as a standard reference to compare the mitochondrial (Dloop1–3, 16S, Nd1, Nd4, COX1, and Cytb) and nuclear DNA (Tert, HK2, Ptger2, and Nduf1) copy number in the cytoplasm of the CON and L+N groups. In the mitochondrial depletion assay, the copy number of each primer for mtDNA and nuclear DNA in the total cell DNA was compared, and the entire nuclear gene was used as an internal reference. All primer sequences are listed in Additional file 1: Table S2.

### Western blotting

The total protein was obtained by lysing tissues or cells in ice-cold RIPA buffer containing phenylmethylsulfonyl fluoride and phosphatase inhibitors for 20 min. Nuclear and Cytoplasmic Protein Extraction Kits (Beyotime, P0027) were used to prepare the nuclear and cytoplasmic proteins, respectively. BCA Protein Assay Kit (Beyotime, P0012) was used to determine the protein concentration. An equal amount of protein was loaded and separated using 10–12% sodium dodecyl-sulfate–polyacrylamide gel electrophoresis (SDS–PAGE) gels (Solarbio, P1200), followed by transfer onto polyvinylidene difluoride membranes. The membranes were washed thrice with Tris-buffered saline with Tween 20 (TBST) following overnight incubation with primary antibody at 4 °C. Then, the membranes were incubated with HRP-conjugated goat anti-rabbit IgG or HRP-conjugated goat anti-mouse IgG (H+L) for 1 h at room temperature. The list of antibodies is as follows: anti-NLRP3 (Abcam, ab270449, 1:1000), anti-ASC (CST, no. 67824, 1:1000), anti-cGAS (CST, no. 31659, 1:1000), anti-STING (Abcam, ab189430, 1:1000), anti-P-STING (CST, no. 72971S, 1:1000), anti-P-IRF3 (CST, no. 29047, 1:1000), anti-IRF3 (CST, no. 4302S, 1:1000), anti-IRF7 (Abcam, ab288440, 1:1000), anti-β-actin (ZSGB-BIO, TA-09, 1:2000), and anti-Lamin B1 (Proteintech, 12987-1-AP, 1:10,000). All experiments were repeated three times independently, and the relative intensities of the protein bands were analyzed using the Image Lab (version 6.1) software.

### Measurement of mitochondrial membrane potential

Mito-Tracker Red CMXRos (Beyotime, C1049B) was used for fluorescent staining of biologically active mitochondria in living cells and to detect mitochondrial membrane potential. Hoechst 33342 staining solution (Beyotime, C1027) was used to stain the nuclei blue, and the nuclei were visualized using fluorescence microscopy after staining for 10 min.

### Cell fluorescence

Following different stimulations, we collected Ana-1 cells and adjusted the concentration to 10^5^–2 × 10^5^/mL. Then, we added 100 µL of cell suspension to a smear and slide centrifuge and centrifuged at 400*g* for 3 min to obtain cell smears. Consequently, we fixed the cells with 4% formaldehyde for 10 min and gently washed them thrice with PBS. Next, the smear was covered with 0.3% Triton X-100 drops for 10 min and blocked with Immunol Staining Blocking Buffer (Beyotime, P0102) for 15 min, followed by overnight incubation with primary antibody at 4 °C. The following day, the cells were washed thrice with PBS and incubated with fluorescent secondary antibody for 1 h in the dark at room temperature. The cells were washed thrice with PBS, and the nuclei were visualized by adding an Antifade Mounting Medium with DAPI (Beyotime, P0131).

### Statistical analysis

Statistical analyses were performed using the GraphPad Prism 9.0 software. Quantitative data are presented as the mean ± standard error of the mean (SEM). One-way analysis of variance (ANOVA) was applied to compare values among the three groups, and a Student’s *t*-test was used for comparison between two groups. A *p*-value < 0.05 was considered statistically significant.

## Results

### cGAS–STING signaling activation in SAP accompanied with increased alveolar macrophage pyroptosis

The disease model was established via the intraperitoneal injection of LPS and CAE in wild-type mice (Fig. 1A). Pathological examination showed pancreatic interlobular septum edema, inflammatory cell infiltration, and apparent structural disorder 24 h after modeling (Fig. 1B). Meanwhile, it showed alveolar edema with congestion and destruction of alveolar structure in the lung tissue (Fig. 1B). Compared with the CON group, the SAP group had increased serum amylase levels and pancreatic tissue injury score, and the rising tissue wet/dry ratio and lung tissue injury score also provided favorable evidence of tissue damage (Fig. 1C–E). The above indicators were consistent with the manifestations of SAP-LI, suggesting that the animal model was successfully established. IL-6 is a reliable indicator of the severity of LI [24]. The elevated serum IL-6 levels in the SAP group were consistent with the pathological manifestations of lung tissue (Fig. 1F).

The NLRP3–ASC–caspase-1 protein complex is one of the most classic inflammasomes that mediate cell pyroptosis. When NLRP3 inflammasomes are activated, caspase-1 cleaves gasdermin D to facilitate its N-terminal domain insertion into the cell membrane and the formation of membrane pores, which eventually induces cell rupture and releases inflammatory mediators [14]. In the lung tissue of SAP mice, the activation of the cGAS–STING signaling pathway was accompanied by an increase in the NLRP3 protein expression levels without significant changes in ASC expression levels. (Fig. 1G and Additional file 2: Fig. S1). These key proteins (cGAS, STING, and NLRP3) had the same expression trend in alveolar macrophages (Additional file 2: Fig. S2). To further explore the pyroptosis of alveolar macrophages, we analyzed the primary macrophages in bronchoalveolar lavage fluid. Flow cytometry analysis revealed a higher proportion of primary alveolar macrophages expressing caspase-1 and PI in the SAP group (Fig. 1H), signifying a notably increased pyroptosis rate of alveolar macrophages compared with the CON group (Fig. 1I). Peritoneal macrophages are crucial in the progression of AP [3, 25]. Similar to alveolar macrophages, peritoneal macrophages in the SAP group exhibited a significantly elevated pyroptosis rate compared with those in the CON group (Additional file 2: Fig. S3). This elevation in the pyroptosis rate may contribute to the promotion of local inflammation and its subsequent systemic spread.

### Downregulation of the cGAS–STING signaling pathway reduces NLRP3 inflammasome activation and macrophage pyroptosis in vivo and in vitro

**Fig. 2:**
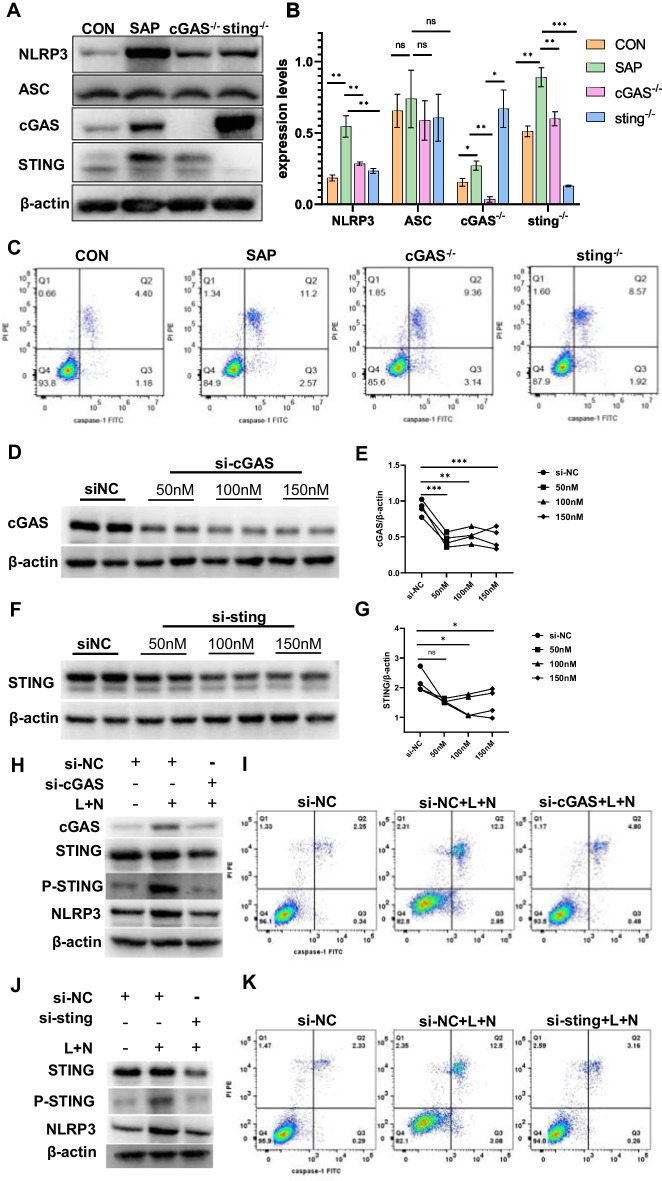
Down-regulation of cGAS–STING signaling pathway reduces the activation of NLRP3 inflammasome and pyroptosis. **A**–**C** Wild type, *cGAS* ^−/−^ and *sting* ^−/−^ mice were injected with CAE and LPS (*n* = 6 per group). Western blot of NLRP3, ASC, cGAS, and STING in lung tissue (**A** ). Protein expression level of **A** (**B**). Flow cytometry was used to detect the pyroptosis of alveolar macrophages ( **C**). **D**–**G** Ana-1 cells were transfected with siRNA (si-cGAS/si-sting) for 48 h. WB and protein expression level of cGAS )**D**, **E**). WB and protein expression level of STING (**F**, **G**). **H**–**K** A total of 48 h after siRNA transfection, Ana-1 cells were stimulated with LPS and nigericin sodium salt. WB of cGAS, STING, P-STING and NLRP3 (**H**). Pyroptosis was detected by flow cytometry (**I**). WB of STING, P-STING and NLRP3 (**J**). Pyroptosis was detected by flow cytometry (**K**). ns, no significance, *p < 0.05, **p < 0.01, ***p < 0.001

To investigate the role of the cGAS–STING signaling pathway in NLRP3 inflammasome-induced pyroptosis, we introduced *cGAS*^*−/−*^ and *sting*^*−/−*^ mice. Pathological examination showed that compared with the SAP group, pancreatic injury and lung injury were significantly reduced after knocking out *cGAS* or *sting* (Additional file 2: Fig. S4). Compared with the CON group, the protein expression levels of NLRP3 in the SAP group were significantly increased. The expression levels of NLRP3 were observed to be downregulated when either *cGAS* or *sting* was knocked out (Fig. 2A, B). It was revealed that the knockout (KO) of *cGAS* or *sting* could improve macrophage pyroptosis, and both alveolar and peritoneal macrophages showed a decrease in the cells double stained with caspase-1 and PI (Fig. 2C and Additional file 2: Figs. S5 and S6).

For the in vitro experiments, we introduced small interfering RNA (siRNA) to knockdown the target genes, and the protein expression levels of cGAS and STING were related to the transfection concentration of siRNA. We found that the best knockdown efficiency was achieved when cells were transfected with 50 nM si-cGAS and 100 nM si-sting (Fig. 2D–G), respectively, and thus, the drug concentration was determined for the subsequent experiments. LPS combined with nigericin was used to induce pyroptosis 48 h after transfection of si-cGAS into Ana-1 cells. In comparison with the si-NC + L+N group, there was a decrease in the expression levels of cGAS, STING, and NLRP3 (Fig. 2H and Additional file 2: Fig. S7), alongside a reduction in macrophage pyroptosis (Fig. 2I and Additional file 2: Fig. S8). A similar pattern was observed following the knockdown of the downstream gene sting (Fig. 2J, K and Additional file 2: Figs. S9 and S10). These findings, in conjunction with in vivo experiments, suggest that knockdown of the cGAS–STING signaling pathway can attenuate macrophage pyroptosis by inhibiting the NLRP3 inflammasome and provide a protective effect for SAP. Notably, in vitro induction of pyroptosis in Ana-1 cells did not raise STING expression levels but did increase STING phosphorylation (Fig. 2J). This phosphorylation could be suppressed by transfection with si-cGAS or si-sting, leading to a subsequent amelioration of pyroptosis.

### Mitochondrial DNA induces pyroptosis by activating cGAS signaling

**Fig.3:**
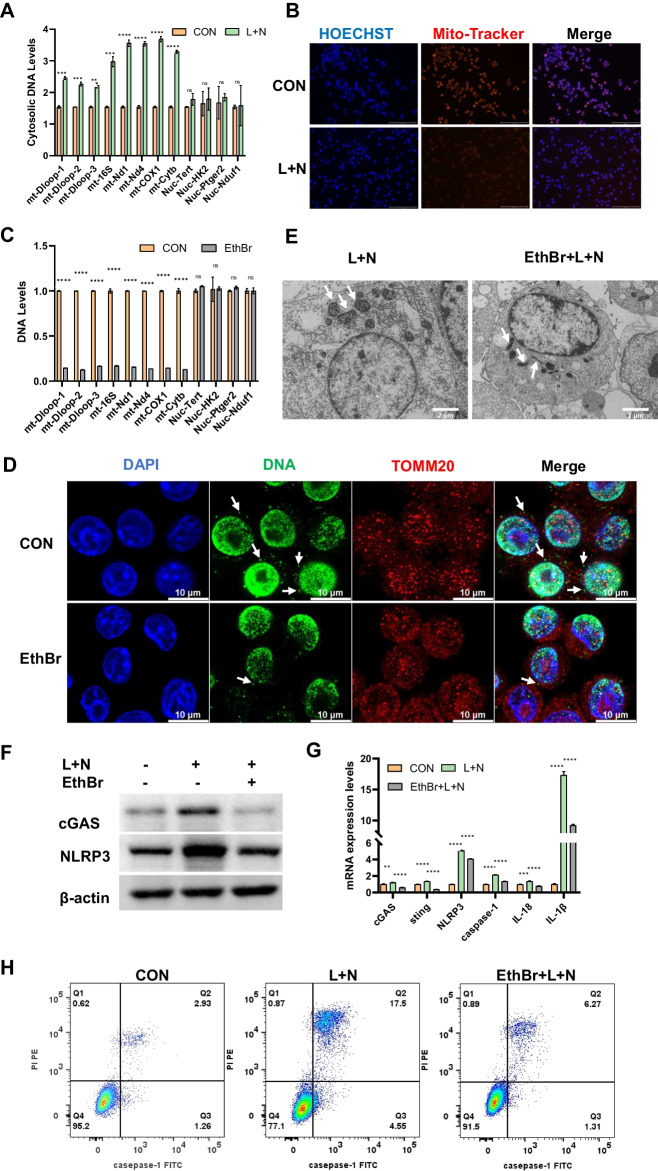
Mitochondrial DNA induces pyroptosis by activating cGAS signaling. **A** Copy number of mitochondrial DNA and nuclear DNA in cytoplasm. **B** Cell fluorescence showing mitochondrial potential, red fluorescence for Mito-Tracker, and blue fluorescence for Hoechst. **C**, **D** Ana-1 cells treated with 100 ng/ml Ethbr for 72 h. Quantification of mitochondrial DNA and nuclear DNA (**C**). DNA and mitochondria were visualized by confocal microscopy, white arrows suggest mitochondrial DNA free from the nucleus (**D**). **E**–**H** A total of 72 h after application of EthBr, LPS, and nigericin sodium salt were administered to induce pyroptosis in Ana-1 cells. Mitochondrial morphology under electron microscope (**E**). The white arrows refer to mitochondria. WB of cGAS and NLRP3 (**F**). **G** mRNA expression levels of cGAS, STING, NLRP3, caspase-1, IL-18, and IL-1β. **H** Pyroptosis of Ana-1 cells was detected by flow cytometry. ns, no significance, *p < 0.05, **p < 0.01, ***p < 0.001, ****p < 0.0001

cGAS recognizes intracellular double-stranded DNA, and several studies have revealed that mtDNA can activate cGAS to induce immune responses and participate in the progression of various diseases [26, 27]. We identified mtDNA as an upstream signal that induces macrophage pyroptosis by activating the cGAS–STING signaling pathway using the mtDNA release and depletion experiment. The copy number of mtDNA in the cytoplasm of Ana-1 cells was significantly higher in the SAP group than that in the CON group following pyroptosis induction. In contrast, the copy number of nuclear DNA in the SAP group was comparable with that in the CON group (Fig. 3A). Meanwhile, we observed that the red fluorescence intensity of the Mito-Tracker in the CON group was significantly higher than that in the L+N group (Fig. 3B), which indicates that the mitochondrial membrane potential significantly decreased after pyroptosis. These results suggested that macrophages were damaged by mitochondrial disruption during pyroptosis, accompanied by a decrease in the membrane potential and leakage of mtDNA.

Then, we cultured Ana-1 cells in a complete medium containing 100 ng/mL EthBr for 72 h. This prolonged exposure to low concentrations of EthBr established a mtDNA-deficient cell line. Compared with the CON group, EthBr-treated Ana-1 cells had a significant reduction in mtDNA copy number, whereas the replication of nuclear DNA was unaffected (Fig. 3C). Confocal microscopy showed that the green fluorescence of DNA free from the nucleus of macrophages induced by EthBr was significantly reduced (Fig. 3D). This reduction in extranuclear double-stranded DNA also supported the inhibitory effect of EthBr on mtDNA. Following the depletion of mtDNA, the standard protocol to induce cell pyroptosis was employed. Transmission electron microscopy images showed that the mitochondria of the L+N group increased in volume and swelled into a round shape. However, the mitochondria of the EthBr + L+N group became dumb-bell or rod shaped, and the mitochondrial swelling was alleviated compared with that of the L+N group (Fig. 3E). EthBr pretreatment notably attenuated the upregulation of cGAS and NLRP3 induced by L+N (Fig. 3F and Additional file 2: Fig. S11), suppressed the transcription of the downstream cGAS gene sting and the pyroptosis-related genes caspase-1, il-18, and il-1β (Fig. 3G). Furthermore, this treatment reduced the macrophage pyroptosis rate from 17.5% to 6.27% (Fig. 3H and Additional file 2: Fig. S12). In conclusion, mtDNA release from damaged mitochondria into the cytosol activates the DNA sensor cGAS, initiating the downstream STING signaling pathway and inducing cell pyroptosis. Pyroptotic cells can further release mtDNA into the cytosol. EthBr’s depletion of mtDNA decreases the upstream signal, thus diminishing the activation of the cGAS–STING pathway. This interruption halts the detrimental cycle of mtDNA–cGAS–STING–pyroptosis–mtDNA, effectively preventing cell death.

### KEGG pathway enrichment analysis of DEGs and identification of hub genes

**Fig. 4:**
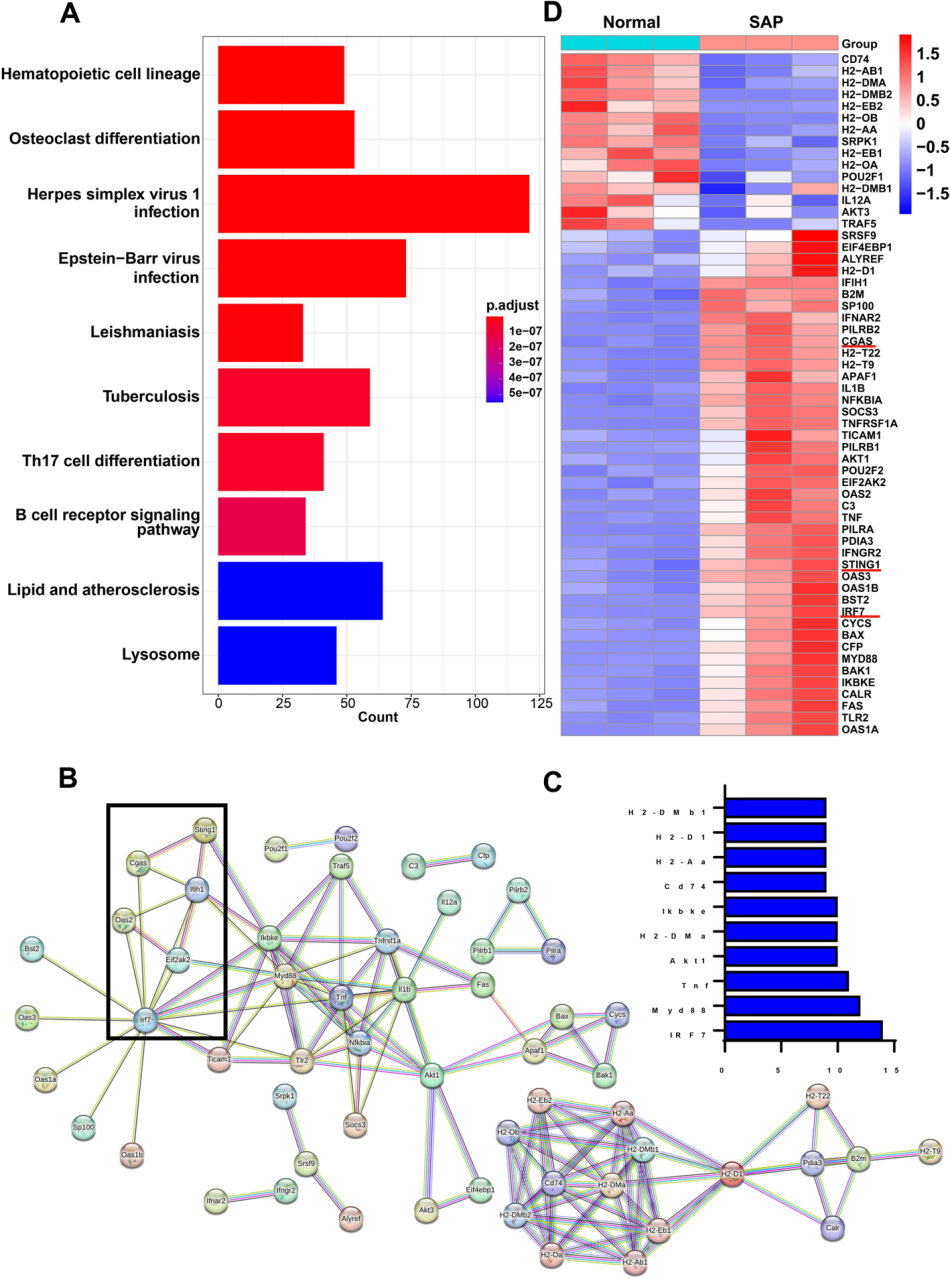
KEGG pathway enrichment analysis of DEGs and identification of hub genes. **A** KEGG pathway for DEGs. **B** The PPI network of genes in herpes simplex virus 1 infection. **C** Top 10 genes with the most junctions. **D** Heat map of DEGs in herpes simplex virus 1 infection pathway

Although activation of the cGAS–STING signaling pathway promotes NLRP3 inflammasome-induced macrophage pyroptosis, the regulatory mechanism of STING on NLRP3 is still unclear. Thus, we decided to analyze the DEGs in the CON and SAP groups to explore the valuable hub genes. KEGG analysis revealed that DEGs were enriched in multiple pathways related to immune function, among which the herpes simplex virus 1 infection pathway had the most DEGs with significant enrichment effects (Fig. 4A). We drew the PPI network for 121 DEGs in this pathway (Fig. 4B). The results showed that multiple genes were interconnected with cGAS and STING, among which IRF7 had the most junctions (up to 14) and was included as a candidate hub gene (Fig. 4C). Following the exclusion of 63 genes with a junction number of zero, the expression levels of the 58 remaining differentially expressed genes (DEGs) were visualized. The heatmap indicated significant upregulation of IRF7 in SAP, aligning with the cGAS–STING pathway trend (Fig. 4D). Additionally, the transcription levels of multiple interferon receptors, specifically IFNAR2 and IFNGR2, were notably higher in the SAP group compared with the CON group (Fig. 4D). IRF7 and IRF3, closely related members of the interferon regulatory factor family, share similar roles in interferon production. Existing studies have demonstrated that STING can enhance IRF3’s regulatory effect on NLRP3, promoting pyroptosis [28, 29]. Given their structural and functional similarities, the involvement of IRF3 and IRF7 in the STING–NLRP3 axis within macrophages was further investigated.

### Downregulation of STING reduced IRF7 and phosphorylated-IRF3 to improve pyroptosis

**Fig. 5:**
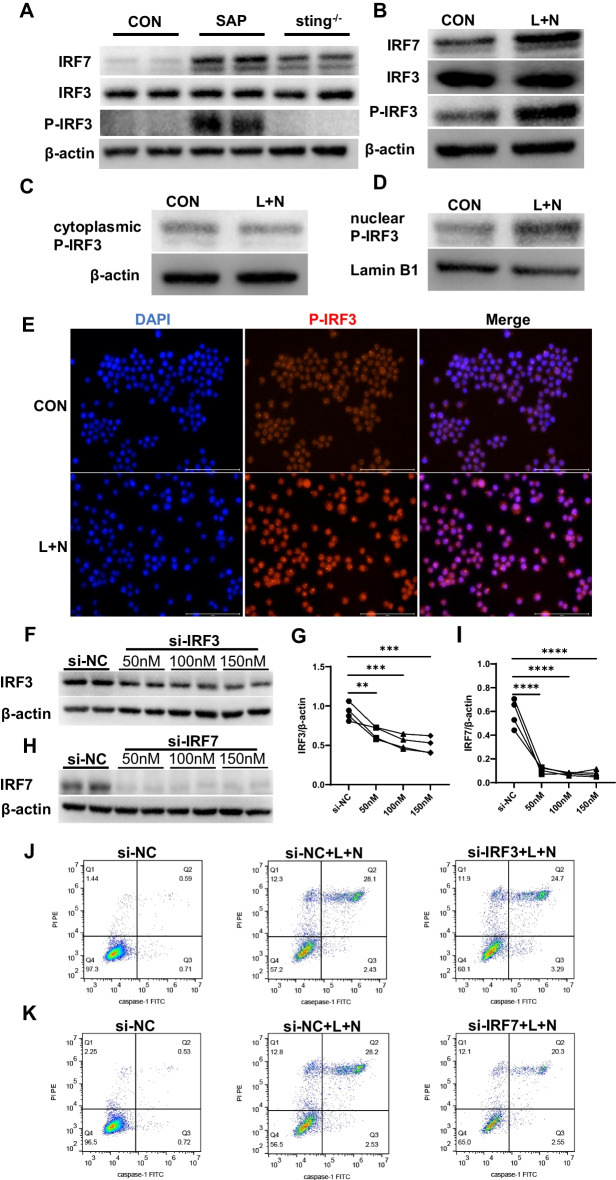
Downregulation of STING reduced IRF7 and P-IRF3 to improve pyroptosis. **A** Wild type mice and *sting* ^−/−^ knockout mice were used to establish SAP animal models in the SAP group and the *sting* ^−/−^ group, respectively. The CON group was injected with normal saline. WB of IRF7, IRF3 and P-IRF3 in alveolar macrophages. **B**–**D** WB of IRF7, IRF3, P-IRF3, cytoplasmic P-IRF3, nuclear P-IRF3. **E** The expression and distribution of P-IRF3 were observed under fluorescence microscope. **F**–**H** Ana-1 cells were transfected with siRNA (si-IRF3/si-IRF7) for 48 h. **F**, **G** WB and protein expression level of IRF3. **H**, **I** WB and protein expression level of IRF7. **J**, **K** A total of 48 h after siRNA transfection, LPS and nigericin sodium salt were used to induce pyroptosis in Ana-1 cells. Pyroptosis was detected by flow cytometry. **p < 0.01, ***p < 0.001, ****p < 0.0001

We induced SAP in wild type and *sting *^*–/–*^ mice and extracted alveolar macrophages for western blot analysis. The results showed that the expression of IRF7 in the SAP group was significantly higher than that in the CON group, which was consistent with the high-throughput sequencing results. Meanwhile, IRF7 was considerably reduced after *sting* knockout (Fig. 5A and Additional file 2: Fig. S13). Although no difference existed in the overall expression of IRF3 among CON, SAP, and sting^–/–^ groups, the phosphorylation level of IRF3 in the SAP group exceeded that in the CON group (Fig. 5A and Additional file 2: Fig. S13). This elevation ceased following the knockdown of sting (Fig. 5A and Additional file 2: Fig. S13). In animal experiments, IRF7 and phosphorylated IRF3 exhibited consistent trends with STING and NLRP3, positively correlating with the level of macrophage pyroptosis.

Comparable outcomes were attained in cell experiments. Following L+N-induced pyroptosis in Ana-1 cells, increased expression of IRF7 and phosphorylated IRF3 (Fig. 5B and Additional file 2: Fig. S14) was accompanied by nuclear translocation of phosphorylated IRF3 (Fig. 5C, D and Additional file 2: Figs S15 and S16). The red fluorescence intensity of the L+N group appeared more pronounced and clustered in the nuclear region under the fluorescence microscope (Fig. 5E). These results were consistent with Li et al. [30] and further confirmed that nuclear translocation of phosphorylated IRF3 was an intermediate link in the STING–NLRP3 axis promoting macrophage pyroptosis.

Subsequently, we transfected si-IRF3 and si-IRF7 into Ana-1 cells and found that 100 nM si-IRF3 and 50 nM si-IRF7 effectively reduced the expression of the target protein (Fig. 5F–I), and thus, both were determined as the experimental concentrations. Pyroptosis was stimulated with L+N 48 h after siRNA transfection. Flow cytometry analysis suggested that knockdown of IRF3 or IRF7 could effectively improve cell pyroptosis induced by L+N, and the pyroptosis rate of macrophages decreased from 28.1% and 28.2% to 24.7% and 20.3% (Fig. 5J, K and Additional file 2: Figs. S17 and S18), respectively.

### Activation of STING upregulates IRF7 and P-IRF3 to mediate NLRP3-induced pyroptosis

**Fig. 6:**
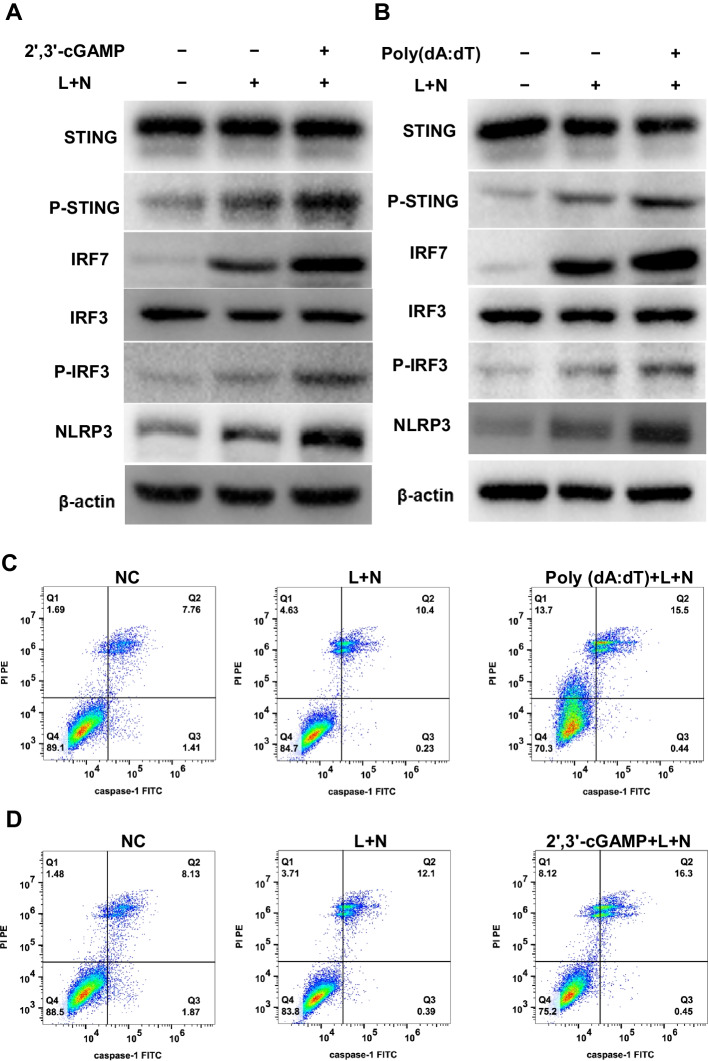
Activation of STING can aggravate pyroptosis by up-regulating IRF7 and P-IRF3. **A**–**D** Ana-1 cells were transfected with 10 ug/ml 2′,3′-cGAMP or 5 ug/mL poly(dA:dT) for 6 h before stimulation with LPS and nigericin sodium salt. Western blot of STING, P-STING, IRF7, IRF3, P-IRF3, and NLRP3 (**A**, **B**). Pyroptosis of Ana-1 cells was measured by flow cytometry (**C**, **D**)

When cGAS detects exogenous or endogenous aberrant double-stranded DNA, it catalyzes the synthesis of 2′,3′-cyclic GMP-AMP (2′,3′-cGAMP) from ATP and GTP, thereby activating STING to initiate the innate immune response [31]. Poly(dA:dT) serves as a double-stranded DNA model with repetitive synthetic sequences of poly(dA-dT):poly(dT-dA). We employed these two inducers from distinct sources to activate STING, followed by the administration of L+N to induce cell pyroptosis. Immunoblotting revealed that early STING stimulation could further enhance STING phosphorylation in response to L+N, simultaneously elevating the expression of IRF7 and P-IRF3, thereby promoting NLRP3 activation (Fig. 6A, B and Additional file 2: Fig. S19 and S20). In comparison to the standard induction regimen (L+N), pretreatment with 2′,3′-cGAMP or poly(dA:dT) notably augmented the pyroptosis of Ana-1 cells, resulting in a greater number of cells stained with caspase-1 and PI (Fig. 6C, D and Additional file 2: Figs. S21 and S22). These findings revealed that STING activation increased the expression of IRF7 and P-IRF3, promoting NLRP3-induced pyroptosis in macrophages.

### Inhibition of IRF7 or IRF3 can alleviate lung injury and macrophage pyroptosis in SAP

**Fig. 7:**
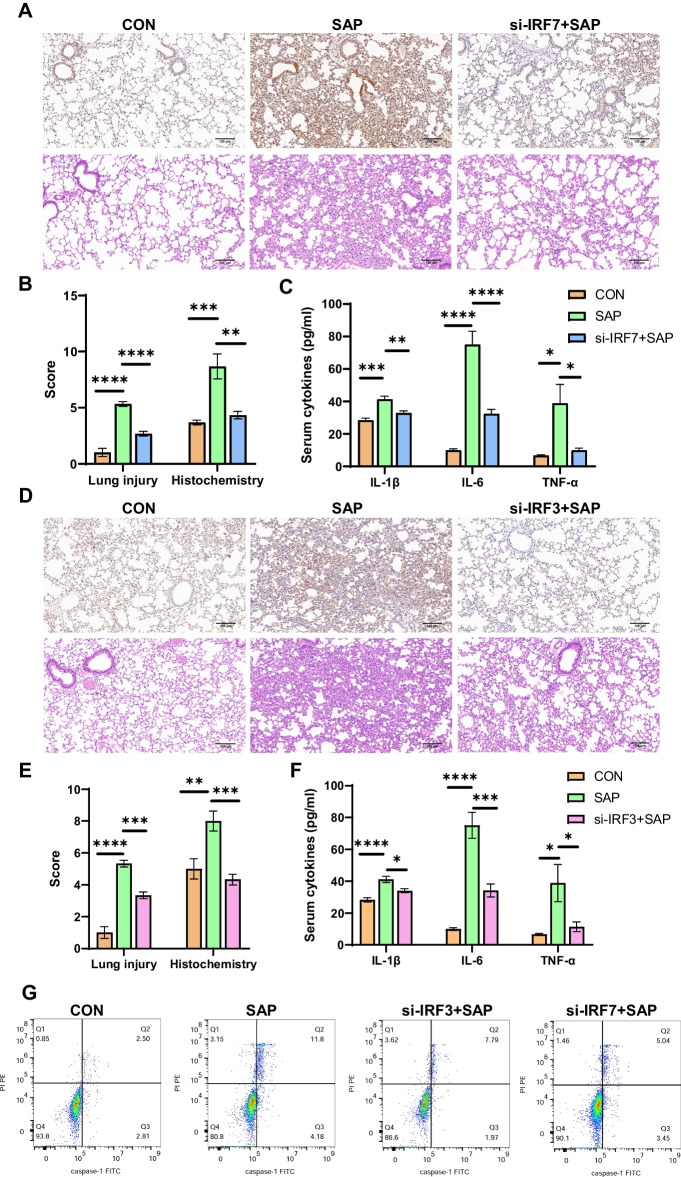
Inhibition of IRF7 or IRF3 can alleviate lung injury and macrophage pyroptosis. **A**–**C** We induced SAP in wild-type mice after tail vein injection of si-IRF7 (*n* = 6 per group). HE staining and IHC of IRF7 in lung tissue (**A**). Score of lung injury and IHC (**B**). The levels of IL-1β, IL-6 and TNF-α in serum (**C**). **D**–**F** We induced SAP in wild-type mice after tail vein injection of si-IRF3 (*n* = 6 per group). HE staining and IHC of IRF3 in lung tissue (**D**). Score of lung injury and IHC (**E**). The levels of IL-1β, IL-6 and TNF-α in serum (**F**). **G** Flow cytometry was used to visualize pyroptosis of alveolar macrophages

We have verified the knockdown efficiency of si-IRF7 and si-IRF3 in cell experiments. According to the manufacturer’s recommendations, we administered tail vein injections of siRNAs for knockdown of IRF7 and IRF3 in mice on day 3 and day 1, respectively, and the mice received intraperitoneal injections of LPS in combination with CAE on day 0 to induce SAP. Immunohistochemistry of lung tissues indicated a diminished staining intensity and reduced extent in the si-IRF7 + SAP and si-IRF3 + SAP groups when compared with the SAP group (Fig. 7A, D), aligning with the protein expression levels (Additional file 2: Fig. S23). In addition, knockdown of IRF7 or IRF3 significantly alleviated pancreatic injury in SAP mice (Additional file 2: Figs. S24). Compared with the SAP group, the atelectasis, pulmonary edema, inflammation, and congestion of the si-IRF7 + SAP and si-IRF3 + SAP groups were improved. The overall pathological scores of LI were decreased (Fig. 7A, B, D, E). Serum cytokine levels were significantly elevated in the SAP group compared with the CON group. Following IRF7 or IRF3 inhibition, the serum levels of IL-6 and TNF-α reduced (Fig. 7C, F), and the systemic inflammation was controlled. The pyroptosis rate of alveolar macrophages decreased from 11.8% to 7.79% and 5.04% after inhibiting IRF7 and IRF3, respectively (Fig. 7G and Additional file 2: Fig. S25), accompanied by a decrease in serum IL-1β levels (Fig. 7C, F). Similarly, pretreatment with si-IRF7 and si-IRF3 can also improve SAP-induced pyroptosis of peritoneal macrophages (Additional file 2: Fig S26). Thus, inhibition of IRF7 or IRF3 can limit the inflammation of SAP and attenuate macrophage pyroptosis and LI, providing a promising therapeutic option with future research prospects.

## Discussion

Acute lung injury (ALI) is one of the most common systemic complications of SAP [32]. About one-third of SAP patients will develop into ALI or even acute respiratory distress syndrome (ARDS) [33], and the mortality of SAP-associated lung injury (SAP-ALI) is as high as 30–40% [33–35]. In AP, macrophages residing in the pancreas are activated first, which is the key to disease initiation [4]. At the same time, macrophages are the most important immune cells migrating to the pancreas, contributing to the amplification of the inflammatory cascade [36]. In the process of local inflammation of pancreatitis progressing to the whole body, peritoneal macrophages are activated by pancreatic enzymes, necrotic substances, inflammatory substances, and even metabolites of pancreatic enzymes digesting fat released into the abdominal cavity by the pancreas [25, 37–39]. Together with Kupffer cells, they release many damaging-related molecular patterns (DAMPS) represented by DNA, HMGB1, and heat shock protein into circulation to mediate the systemic inflammatory response [40–43]. Several studies have found that alveolar macrophages can accept the stimulation of circulating inflammatory mediators to participate in the inflammatory response of ALI [44–46]. Therefore, ALI is the consequence of SAP spreading to the whole body and is closely related to the sequential activation of macrophages.

In this study, we demonstrate the protein expression levels of cGAS, STING, and NLRP3 in SAP and detected the pyroptosis of alveolar and peritoneal macrophages. In addition, we used gene knockout mice to knockdown *cGAS* and *sting* and observed that downregulation of cGAS–STING signaling can reduce NLRP3 inflammasome-induced macrophage pyroptosis and ameliorate LI in SAP. Meanwhile, in vitro experiments using siRNA knockdown of target genes verified the regulatory effect of the cGAS–STING signaling pathway on the NLRP3–pyroptosis axis. The above experimental results were consistent with previous studies on NLRP3 inflammasome activation induced by cGAS–STING [20, 47, 48].

The cGAS–STING signaling is prominently activated in macrophages in the context of SAP. Zhao Q et al. have found that macrophages in the pancreas were the predominant source of leukocyte STING expression [15]. STING can induce NLRP3-dependent pyroptosis of macrophages to secrete IL-1β and IL-18, and the abnormal activation of zymogens granules in macrophages can promote the production of TNF-α, IL-6, IL-1β, and other inflammatory cytokines by upregulating the NF-κB signaling pathway [5]. In addition, NF-κB, as a broad transcription factor, can promote the transcription of NLRP3 and is regulated by STING signaling [49–52]. Therefore, STING in macrophages may be a vital signaling molecule mediating pancreatic injury in AP.

The cGAS–STING signaling pathway can recognize exogenous DNA released from viruses, bacteria, or damaged cells and self-damaged genomic or mtDNA, thus performing various innate immune defense functions [53, 54]. Mitochondrial damage is an important pathogenic factor of acute LI [55, 56]. Mitochondrial dysfunction and mtDNA release, accompanied with a large number of reactive oxygen species (ROS) generation, can be observed in various types of LI [57]. Studies have revealed that maintaining the dynamic balance of mitochondrial fusion/fission is conducive to improving LI caused by endotoxins in vivo or in vitro [58]. Histone deacetylase 3 deficiency alleviates sepsis-induced acute LI by maintaining mitochondrial quality control through the FOXO1–ROCK1 axis [59]. mtDNA and mtROS are necessary for activating NLRP3 inflammasomes [60, 61], and it has been found that newly synthesized mtDNA binds to NLRP3 and is associated with its activation during mitochondrial damage [62]. Oxidized mitochondrial DNA leaks into the cytoplasm through mitochondrial permeability transition pore- and voltage-dependent anion channel (VDAC)-dependent channels, initiating NLRP3 inflammasome activation [63]. Meanwhile, the inhibition of VDAC can inhibit NLRP3 inflammasome activation and ROS production by reducing mitochondrial activity [64]. In our experiments, we found that cell pyroptosis significantly increased mtDNA leakage from the mitochondria into the cytoplasm. When mtDNA is depleted, the cytoplasm’s free double-stranded DNA decreases, and cGAS signaling is significantly weakened, accompanied by downregulation of cell pyroptosis. Therefore, as an upstream molecule of cGAS, mtDNA is involved in NLRP3-induced macrophage pyroptosis by activating the cGAS–STING signaling pathway.

However, the molecular regulatory mechanism connecting the mtDNA–cGAS–STING axis to the NLRP3–pyroptosis axis necessitates further investigation. To delve deeper, we conducted high-throughput sequencing and bioinformatics analysis on blood samples obtained from normal and SAP mice. The results pointed towards IRF7 exhibiting the highest number of junctions among the DEGs, with its transcript level significantly higher in the SAP group compared with the CON group. Animal experiments corroborated these sequencing findings, as STING knockdown notably attenuated the increased expression of IRF7 induced by SAP. As downstream signaling molecules of STING, IRF3 and IRF7 frequently share overlapping immune functions, particularly in regulating the production of type I interferon [65]. These molecules exhibit transcriptional activity within a phase-separated state, and their intracellular localization holds significance in innate immunity [66]. Therefore, we aim to investigate the role of the two proteins in macrophage pyroptosis activated by SAP.

IRF3 can be phosphorylated by TANK-binding kinase-1 (TBK1) to participate in STING-mediated inflammatory and immune responses and facilitate the progression of inflammatory and infectious diseases by regulating the transcription of interferons and inflammatory factors [67–69]. Positive regulation of IRF3-mediated type I interferon can limit pancreatic injury caused by coxsackievirus B3 replication [70]. Wip1 aggravates CAE-induced autophagy and inflammatory injury in acinar cells by targeting STING/TBK1/IRF3 in experimental pancreatitis [71]. Knockout of IRF3 in animal experiments alleviated multiple cell death modes, including apoptosis, necrosis, and pyroptosis [72]. In the current study, it was observed that STING knockout significantly decreased the expression of IRF7 and the nuclear translocation of phosphorylated IRF3 in macrophages from SAP mice. Downregulating IRF7 or IRF3 produced similar experimental outcomes as STING knockout, both of which contributed to ameliorating drug-induced macrophage pyroptosis. We administered 2′,3′-cGAMP and poly (dA:dT) to simulate the biological process of STING activating IRF3 and IRF7 in vivo and in vitro, and found that premature activation of IRF3 or IRF7 exacerbated macrophage pyroptosis. Previous studies have indicated that reducing the STING-IRF3 signaling pathway can help mitigate NLRP3-induced pyroptosis [29]. The STING signal can influence the transcriptional level of NLRP3 by modulating the binding strength between IRF3 and the NLRP3 promoter region [28]. These findings align with our results, demonstrating that STING signaling activation promotes IRF3 phosphorylation and translocation into the nucleus, thereby accelerating NLRP3-induced cell pyroptosis. Unfortunately, we did not perform further experiments to verify the mechanism by which P-IRF3 regulates NLRP3 transcription.

Activation of IRF7 strengthens innate immunity and facilitates the control of bacterial and viral infections, which is prevalent in inflammatory and infectious diseases. IRF7 activation has been found to aggravate autoimmune pancreatitis, inflammatory progression, and fibrosis in systemic sclerosis [73–75]. A recent study found that IRF7 is involved in the progression of sepsis associated LI by regulating Srg3 and ferroptosis [76], but there are no reports on the mechanism of IRF7 in SAP. Our experiment found that the knockdown of IRF7 could reduce pyroptosis and LI induced by SAP, revealing, for the first time, the link between IRF7 and macrophage pyroptosis in SAP. Unfortunately, there is no significant change in the phosphorylation level or nucleoplasmic distribution of IRF7 before and after macrophage pyroptosis, and the specific mechanism of IRF7 regulating NLRP3 activation still needs to be clarified. A study found that IRF7 may promote pyroptosis by regulating the promoter of NLRP3 in the nerve cells [77]. MiR-30b-5p, originating from M2 macrophage exosomes, has the ability to inhibit pyroptosis in airway epithelial cells, thereby improving the progression of asthma through its targeting of IRF7 [78]. In a study by Bin Gong et al., it was observed that silencing IRF7 led to the amelioration of chondrocyte pyroptosis and inflammation in osteoarthritis, possibly through its interaction with FGF21. In oncological contexts, IRF7 acts as an inhibitor of pyruvate kinase M2 (PKM2) transcription, consequently downregulating the Warburg effect and influencing the malignancy of osteosarcoma [79]. The reduction of PKM2 can induce pyroptosis in esophageal squamous carcinoma cells, providing further evidence of IRF7’s involvement in the regulation of cell pyroptosis [80].

Many previous experiments have demonstrated that cell pyroptosis promotes the development of acute pancreatitis [81], and multiple drugs exert protective effects in acute pancreatitis by inhibiting pyroptosis-related signaling pathways [12, 13]. So, can inhibiting STING or NLRP3 ameliorate pancreatic and lung damage caused by pancreatitis? Our study found that either knocking out STING or silencing its expression by siRNA can downregulate NLRP3 and improve pancreatic injury and lung injury. STING inhibitor C-176 can also provide protection against AP [15, 22]. Studies have found that emodin can reduce the increased expression of NLRP3, ASC, caspase-1 p10, and GSDMD proteins in alveolar macrophages induced by acute pancreatitis and improve pancreatitis-associated lung injury by inhibiting macrophage pyroptosis pathway [82, 83]. Exosomes derived from plasma of AP mice can trigger NLRP3 inflammasome-dependent pyroptosis in alveolar macrophages. Inhibiting the release or uptake of exosomes with inhibitors can significantly inhibit alveolar macrophage pyroptosis, thereby alleviating pancreatitis-induced lung injury [84]. Pyroptosis is a double-edged sword [85]. Inhibition of pyroptosis can down-regulate inflammatory response, hinder immune cell infiltration, and adversely affect antitumor and anti-infection immunity. Therefore, when formulating anti-pyroptosis strategies for pancreatitis, we need to consider multiple tissues and organs throughout the body, consider the long-term effects of intervention measures, and develop treatment and research methods suitable for patients with AP with a cautious attitude.

**Fig. 8:**
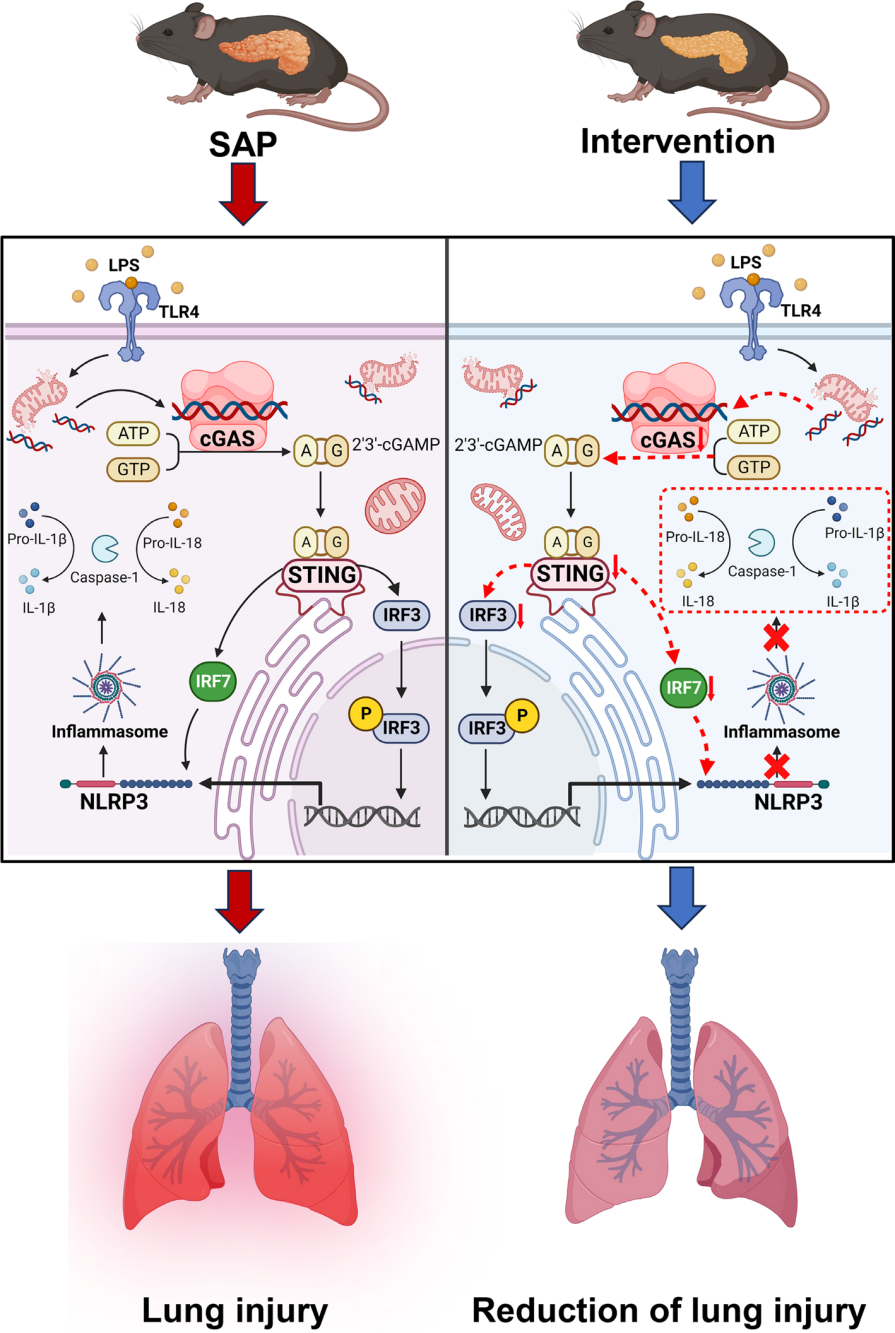
The mechanism diagram shows that IRF7 and IRF3 can serve as intermediate signals for STING and NLRP3, linking the mtDNA–cGAS–STING axis and NLRP3–pyrotosis axis to further explain the molecular mechanism by which STING regulates the activation of NLRP3 inflammasomes. Inhibiting multiple molecules from mtDNA to NLRP3 can reduce macrophage pyroptosis and improve SAP-ALI

This study demonstrates that macrophages become activated in SAP, leading to the release of mtDNA from damaged mitochondria into the cytosol (Fig. 8 left). The mtDNA–cGAS–STING axis mediates NLRP3 inflammasome-induced pyroptosis by upregulating phosphorylated IRF3 entry into the nucleus and increasing IRF7 expression levels. Various interventions, such as mitochondrial depletion, knockout of *cGAS* or *sting*, and inhibition of IRF3 or IRF7 expression, can interfere with the activation of NLRP3 inflammasome (Fig. 8 right), thereby improving the severity of macrophage pyroptosis and SAP-LI. These results provide strong evidence and potential targets for antipyroptosis therapy with clinical application prospects in AP.

### Supplementary Information

**Additional file 1: Table S1.** The sequences of siRNAs. **Table S2.** The sequences of the primers

**Additional file 2: Fig. S1.** The protein expression levels of Fig. 1G. **Fig. S2.** The protein expression levels in alveolar macrophages. **Fig. S3.** The pyroptosis of peritoneal macrophages. **Fig. S4.** Pathological manifestations and scores of pancreatic and lung tissues. **Fig. S5.** The pyroptosis rate of alveolar macrophages in Fig. 2C. **Fig. S6.** The pyroptosis of peritoneal macrophages. **Fig. S7.** The protein expression levels of Fig. 2H. **Fig. S8.** The pyroptosis rate of Fig. 2J. **Fig. S9.** The protein expression levels of Fig. 2J. **Fig. S10.** The pyroptosis rate of Fig. 2K. **Fig. S11.** The protein expression levels of Fig. 3F. **Fig. S12.** The pyroptosis rate of Fig. 3H. **Fig. S13.** The protein expression levels of Fig. 5A. **Fig. S14.** The protein expression levels of Fig. 5B. **Fig. S15.** The protein expression levels of Fig. 5C. **Fig. S16.** The protein expression levels of Fig. 5D. **Fig. S17.** The pyroptosis rate of Fig. 5J. **Fig. S18.** The pyroptosis rate of Fig. 5K. **Fig. S19.** The protein expression levels of Fig. 6A. **Fig. S20.** The protein expression levels of Fig. 6B. **Fig. S21.** The pyroptosis rate of Fig. 6C. **Fig. S22.** The pyroptosis rate of Fig. 6D. **Fig. S23.** The protein expression levels of IRF7, IRF3, and NLRP3. **Fig. S24.** Pathological manifestations and scores in pancreas. **Fig. S25.** The pyroptosis rate of alveolar macrophages in Fig. 7G. **Fig. S26.** The pyroptosis of peritoneal macrophages.

## Data Availability

The datasets used and/or analyzed during the current study are available from the corresponding author on reasonable request.

## Abbreviations

mtDNA: Mitochondrial DNA
cGAS: Cyclic GMP–AMP synthase
STING: Stimulator of interferon genes
IRF7: Interferon regulatory factor 7
IRF3: Interferon regulatory factor 3
SAP: Severe acute pancreatitis
LI: Lung injury
NLRP3: NOD-, LRR-, and pyrin domain-containing protein 3
IL-6: Interleukin 6
IL-1β: Interleukin 1β
TNF-α: Tumor necrosis factor α
SAP-ALI: Severe acute pancreatitis-associated lung injury
AP: Acute pancreatitis
PAMPs: Pathogen-associated molecular patterns
DAMPs: Damage-associated molecular patterns
ASC: Apoptosis-associated speck-like protein containing a CARD
CAE: Caerulein
LPS: Lipopolysaccharide
EthBr: Ethidium bromide
DEGs: Differentially expressed genes
KEGG: Kyoto Encyclopedia of Genes and Genomes
PPI: Protein–protein interaction
GEO: Gene expression omnibus
IHC: Immunohistochemistry
HRP: Horseradish peroxidase
KO: Knockout
2ʹ,3ʹ-cGAMP: 2ʹ,3ʹ-Cyclic GMP-AMP
VODC: Voltage-dependent anion channel
TBK1: TANK-binding kinase-1
PKM2: Pyruvate kinase M2
