# Supplementary material for: Mitochondrial (mt)DNA–cyclic GMP–AMP synthase (cGAS)–stimulator of interferon genes (STING) signaling promotes pyroptosis of macrophages via interferon regulatory factor (IRF)7/IRF3 activation to aggravate lung injury during severe acute pancreatitis
Source: Cell Mol Biol Lett. 2024 Apr 27;29:61. doi: 10.1186/s11658-024-00575-9 (PMC11055249; doi:10.1186/s11658-024-00575-9)
Supplement: Supplementary file 1 — Additional file 1: Table S1. The sequences of siRNAs. Table S2. The sequences of the primers [file 11658_2024_575_MOESM1_ESM.docx]

| Table S1. The sequences of siRNAs | |
| --- | --- |
| siRNA | sequence |
| si-cGAS | CCAAGATGCTGTCAAAGTT |
| si-sting | CCATGTCACAGGATGCCAA |
| si-IRF7 | GCACTACACAGAGACGCTT |
| si-IRF3 | CCTCAGATCTGGCTATTGT |

| Table S2. The sequences of the primers | |
| --- | --- |
| primer | sequence |
| cGAS | F: 5'-CAGGAAGGAACCGGACAAGC-3' |
|  | R: 5'-CCGACTCCCGTTTCTGCATT-3' |
| sting | F: 5'-TCGCACGAACTTGGACTACTG-3' |
|  | R: 5'-CCAACTGAGGTATATGTCAGCAG-3' |
| NLRP3 | F: 5'-ATCAACAGGCGAGACCTCTG-3' |
|  | R: 5'-GTCCTCCTGGCATACCATAGA-3' |
| caspase-1 | F: 5'-ACAAGGCACGGGACCTATG-3' |
|  | R: 5'-ACAAGGCACGGGACCTATG-3' |
| IL-18 | F: 5'-GTGAACCCCAGACCAGACTG-3' |
|  | R: 5'-GTGAACCCCAGACCAGACTG-3' |
| IL-1β | F: 5'-GAAATGCCACCTTTTGACAGTG-3' |
|  | R: 5'-TGGATGCTCTCATCAGGACAG-3' |
| β-actin | F: 5'-GGCTGTATTCCCCTCCATCG-3' |
|  | R: 5'-CCAGTTGGTAACAATGCCATGT-3' |
| mt-Dloop-1 | F: 5'-AATCTACCATCCTCCGTGAAACC-3' |
|  | R: 5'-TCAGTTTAGCTACCCCCAAGTTTAA-3' |
| mt-Dloop-2 | F: 5'-CCCTTCCCCATTTGGTCT-3' |
|  | R: 5'-TGGTTTCACGGAGGATGG-3' |
| mt-Dloop-3 | F: 5'-TCCTCCGTGAAACCAACAA-3' |
|  | R: 5'-AGCGAGAAGAGGGGCATT-3' |
| mt-16S | F: 5'-CACTGCCTGCCCAGTGA-3' |
|  | R: 5'-ATACCGCGGCCGTTAAA-3' |
| mt-Nd1 | F: 5'-CTAGCAGAAACAAACCGGGC-3' |
|  | R: 5'-CCGGCTGCGTATTCTACGTT-3' |
| mt-Nd4 | F: 5'-AACGGATCCACAGCCGTA-3' |
|  | R: 5'-AGTCCTCGGGCCATGATT-3' |
| mt-COX1 | F: 5'-GCCCCAGATATAGCATTCCC-3' |
|  | R: 5'-GTTCATCCTGTTCCTGCTCC-3' |
| mt-Cytb | F: 5'-GCTTTCCACTTCATCTTACCATTTA-3' |
|  | R: 5'-TGTTGGGTTGTTTGATCCTG-3' |
| nuc-Tert | F: 5'-CTAGCTCATGTGTCAAGACCCTCTT-3' |
|  | R: 5'-GCCAGCACGTTTCTCTCGTT-3' |
| nuc-HK2 | F: 5'-GCCAGCCTCTCCTGATTTTAGTGT-3' |
|  | R: 5'-GGGAACACAAAAGACCTCTTCTGG-3' |
| nuc-Ptger2 | F: 5'-CCTGCTGCTTATCGTGGCTG-3' |
|  | R: 5'-GCCAGGAGAATGAGGTGGTC-3' |
| nuc-Nduf1 | F: 5'-CTTCCCCACTGGCCTCAAG-3' |
|  | R: 5'-CCAAAACCCAGTGATCCAGC-3' |
