# Supplementary material for: Mitochondrial (mt)DNA–cyclic GMP–AMP synthase (cGAS)–stimulator of interferon genes (STING) signaling promotes pyroptosis of macrophages via interferon regulatory factor (IRF)7/IRF3 activation to aggravate lung injury during severe acute pancreatitis
Source: Cell Mol Biol Lett. 2024 Apr 27;29:61. doi: 10.1186/s11658-024-00575-9 (PMC11055249; doi:10.1186/s11658-024-00575-9)
Supplement: Supplementary file 2 — Additional file 2: Fig. S1. The protein expression levels of Fig. 1G. Fig. S2. The protein expression levels in alveolar macrophages. Fig. S3. The pyroptosis of peritoneal macrophages. Fig. S4. Pathological manifestations and scores of pancreatic and lung tissues. Fig. S5. The pyroptosis rate of alveolar macrophages in Fig. 2C. Fig. S6. The pyroptosis of peritoneal macrophages. Fig. S7. The protein expression levels of Fig. 2H. Fig. S8. The pyroptosis rate of Fig. 2J. Fig. S9. The protein expression levels of Fig. 2J. Fig. S10. The pyroptosis rate of Fig. 2K. Fig. S11. The protein expression levels of Fig. 3F. Fig. S12. The pyroptosis rate of Fig. 3H. Fig. S13. The protein expression levels of Fig. 5A. Fig. S14. The protein expression levels of Fig. 5B. Fig. S15. The protein expression levels of Fig. 5C. Fig. S16. The protein expression levels of Fig. 5D. Fig. S17. The pyroptosis rate of Fig. 5J. Fig. S18. The pyroptosis rate of Fig. 5K. Fig. S19. The protein expression levels of Fig. 6A. Fig. S20. The protein expression levels of Fig. 6B. Fig. S21. The pyroptosis rate of Fig. 6C. Fig. S22. The pyroptosis rate of Fig. 6D. Fig. S23. The protein expression levels of IRF7, IRF3, and NLRP3. Fig. S24. Pathological manifestations and scores in pancreas. Fig. S25. The pyroptosis rate of alveolar macrophages in Fig. 7G. Fig. S26. The pyroptosis of peritoneal macrophages. [file 11658_2024_575_MOESM2_ESM.pdf]

Fig. S1

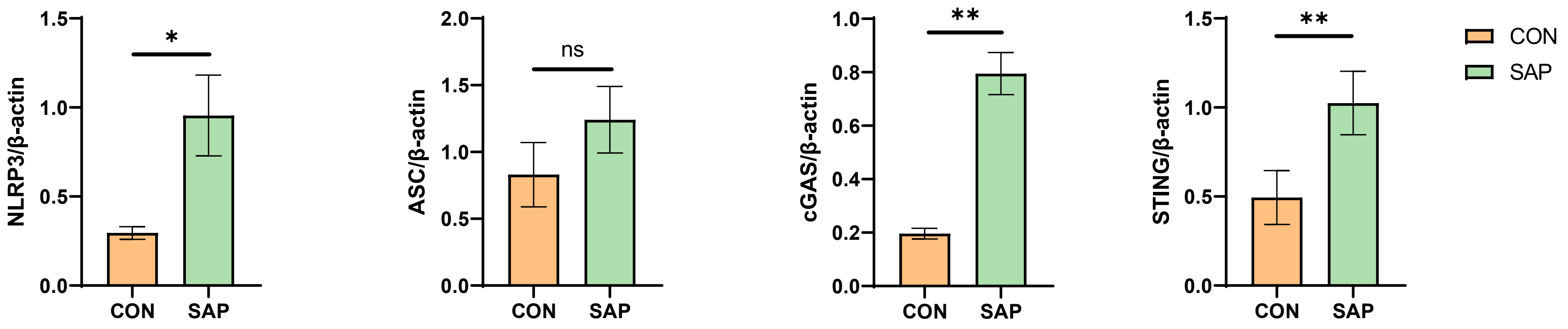

NLRP3/β-actin
ASC/β-actin
cGAS/β-actin
STING/β-actin
*
ns
**
**
CON
SAP

Fig. S2

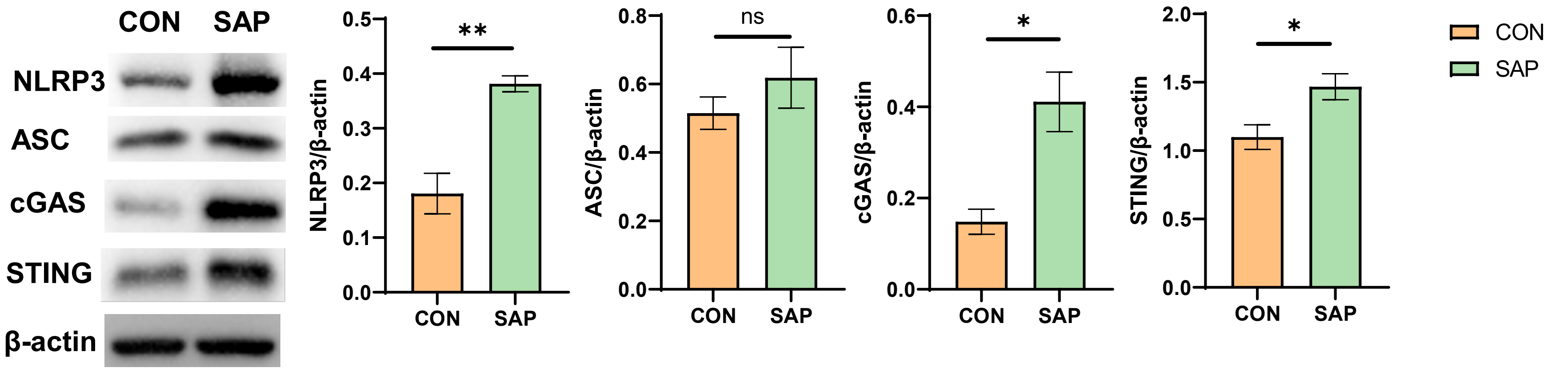

CON
SAP
NLRP3
ASC
cGAS
STING
β-actin
NLRP3/β-actin
ASC/β-actin
cGAS/β-actin
STING/β-actin
**
ns
*
*

Fig. S3

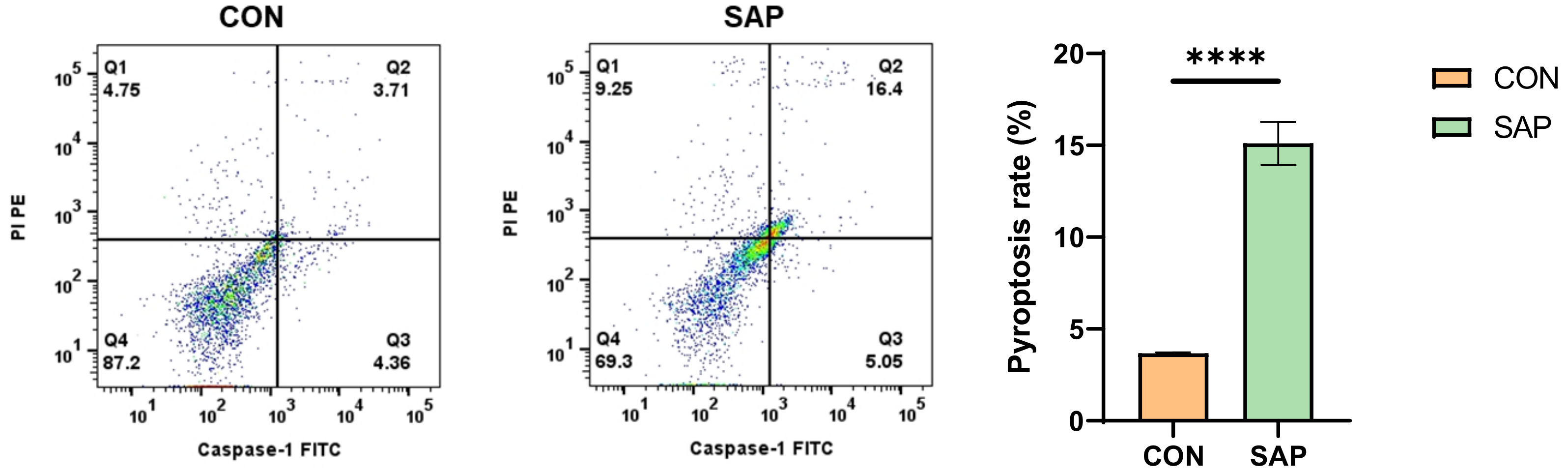

CON
SAP
Q1
4.75
Q2
3.71
Q4
87.2
Q3
4.36
Q1
9.25
Q2
16.4
Q4
69.3
Q3
5.05
PI PE
Caspase-1 FITC
Pyroptosis rate (%)
****
CON
SAP

Fig. S4

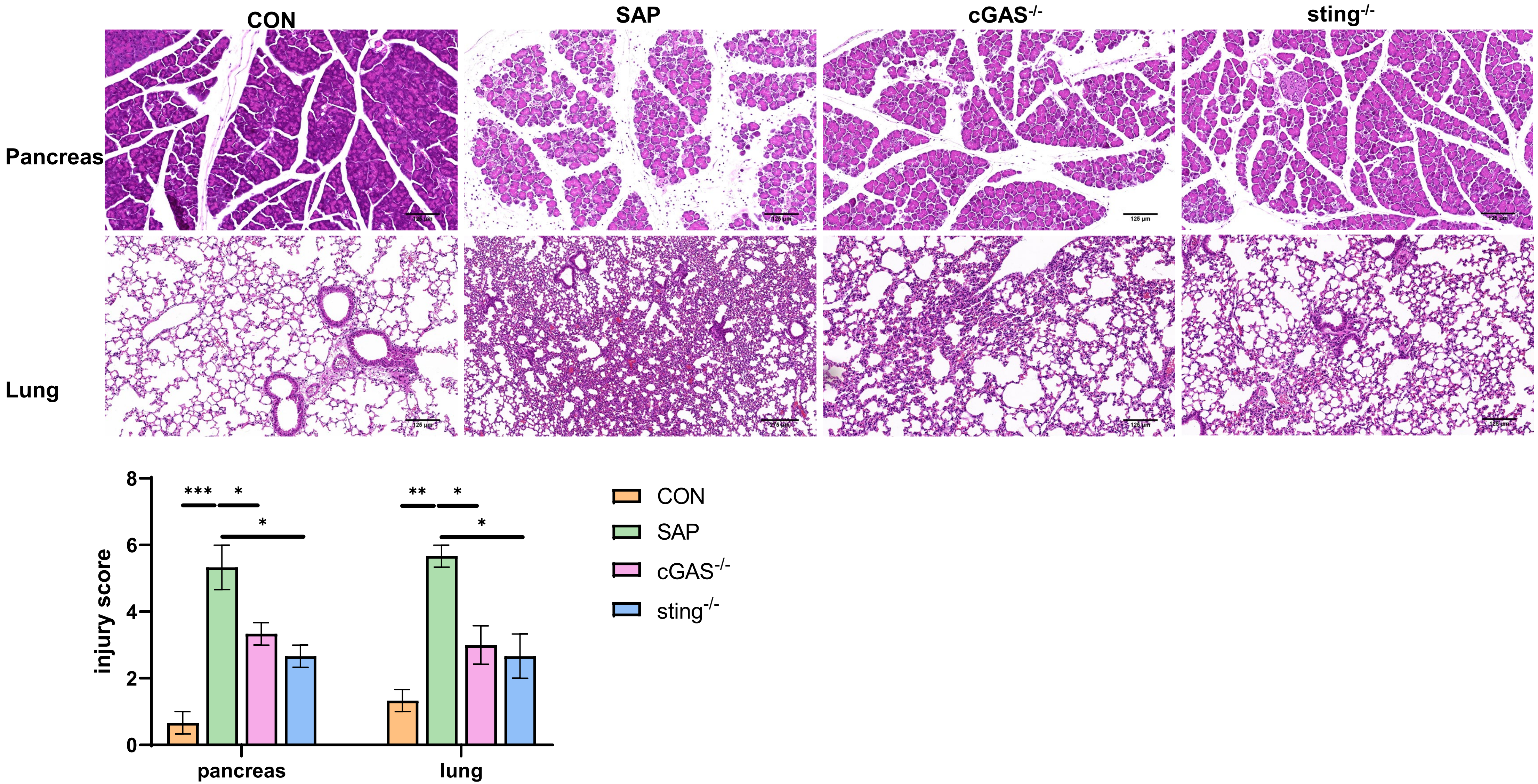

CON
SAP
cGAS-/-
sting-/-
Pancreas
Lung
125 μm
injury score
8
6
4
2
0
***
**
*
pancreas
lung
CON
SAP
cGAS-/-
sting-/-

Fig. S5

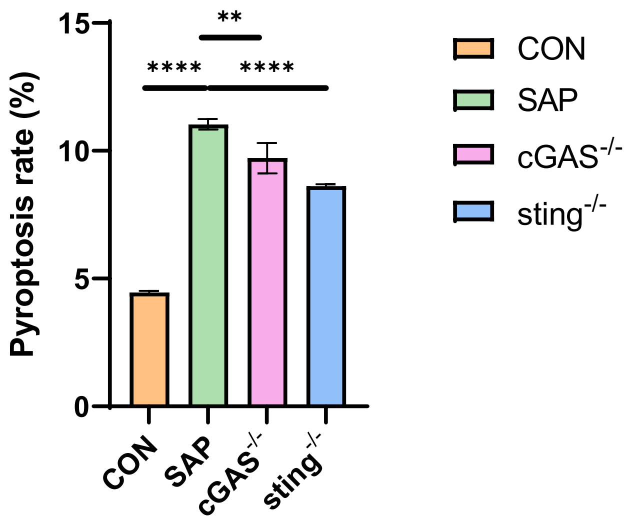

Pyroptosis rate (%)
15
10
5
0
**
****
****
CON
SAP
cGAS-/-
sting-/-
CON
SAP
cGAS-/-
sting-/-

Fig. S6

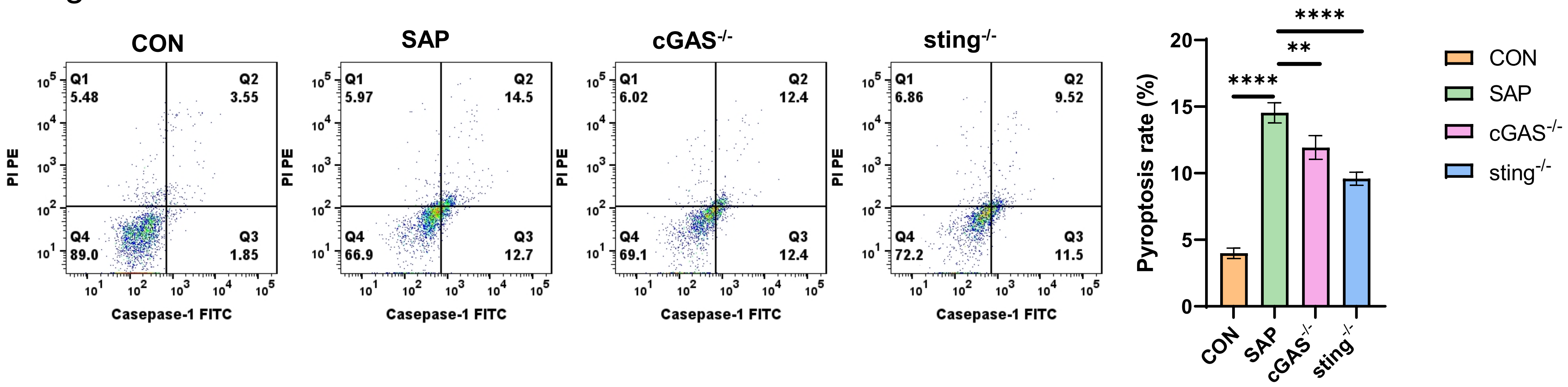

CON
SAP
cGAS-/-
sting-/-
Q1 5.48
Q2 3.55
Q4 89.0
Q3 1.85
Q1 5.97
Q2 14.5
Q4 66.9
Q3 12.7
Q1 6.02
Q2 12.4
Q4 69.1
Q3 12.4
Q1 6.86
Q2 9.52
Q4 72.2
Q3 11.5
PI PE
Casepase-1 FITC
Pyroptosis rate (%)
20
15
10
5
0
****
**
****
CON
SAP
cGAS-/-
sting-/-

Fig. S7

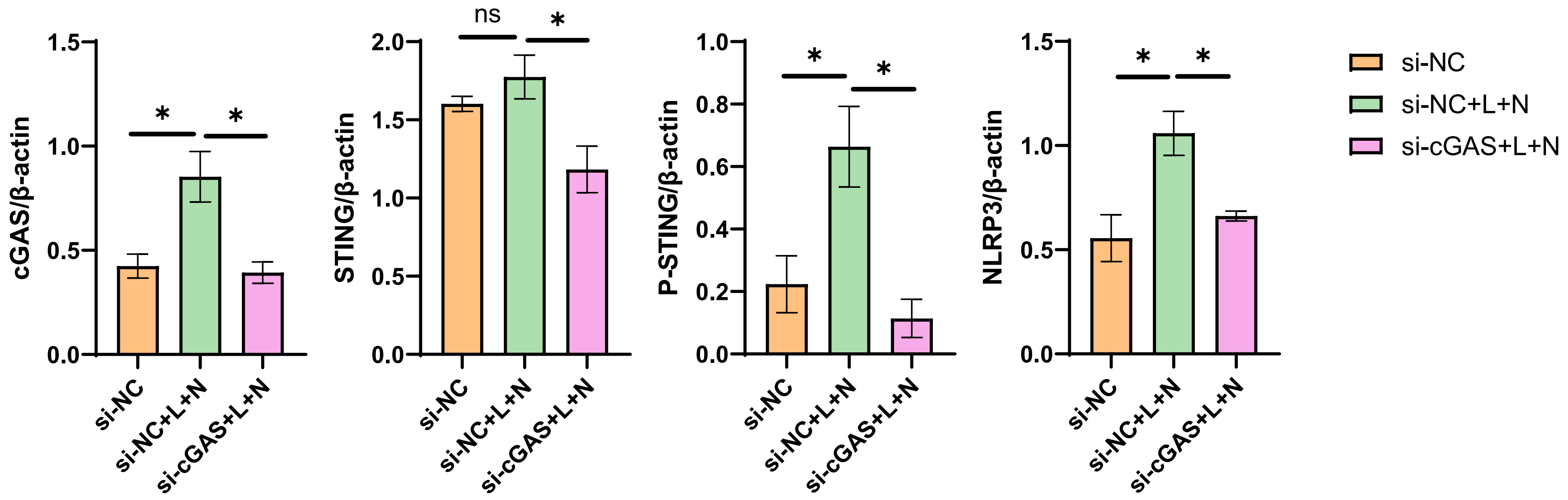
cGAS/β-actin
STING/β-actin
P-STING/β-actin
NLRP3/β-actin
ns
*
si-NC
si-NC+L+N
si-cGAS+L+N

Fig. S8

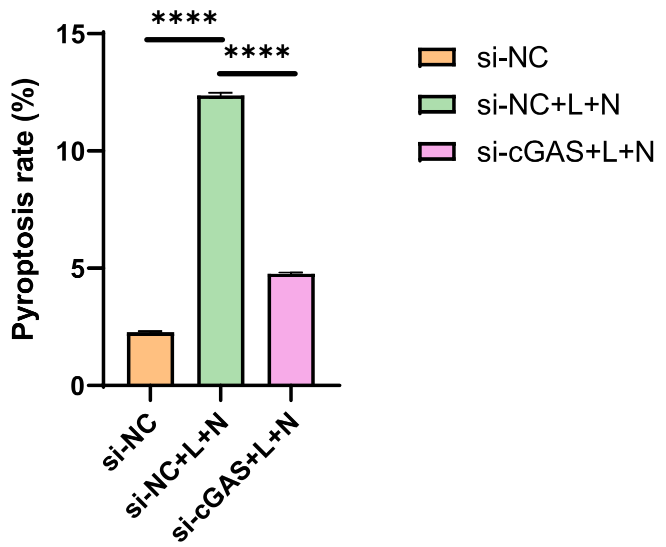
Pyroptosis rate (%)
****
si-NC
si-NC+L+N
si-cGAS+L+N

Fig. S9

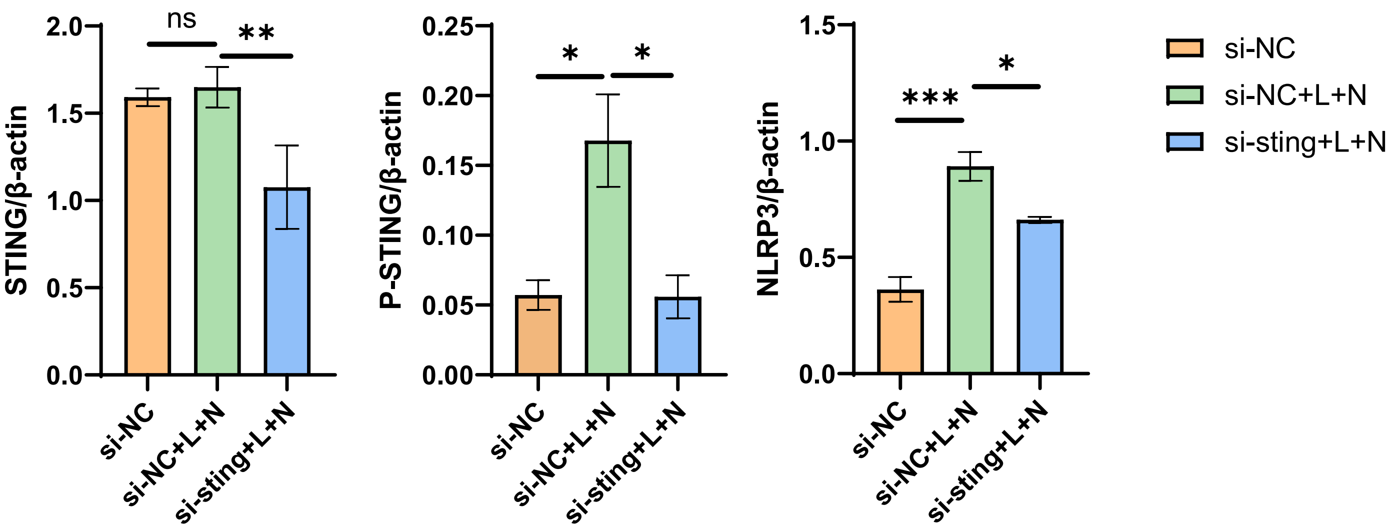

Fig. S10

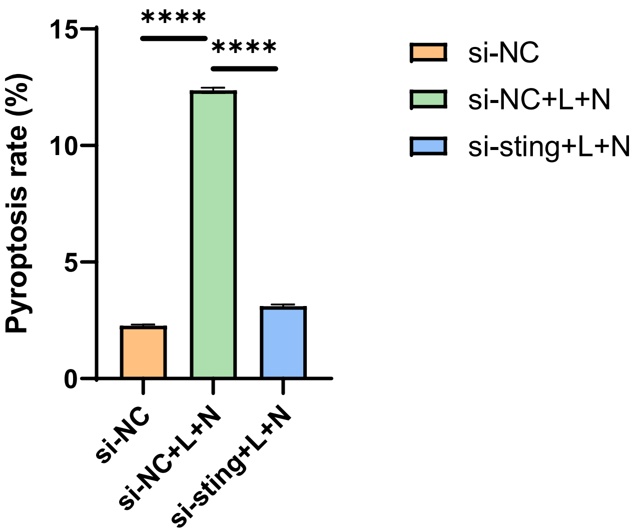

Fig. S11

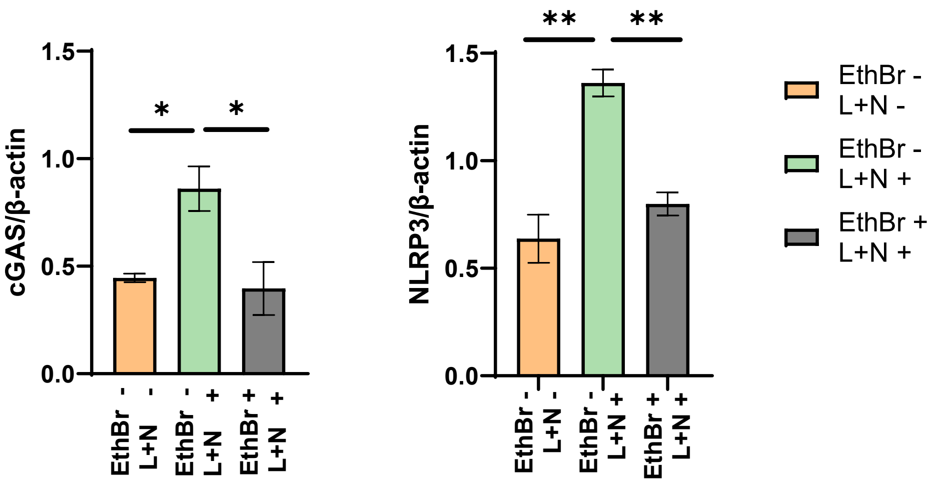

Fig. S12

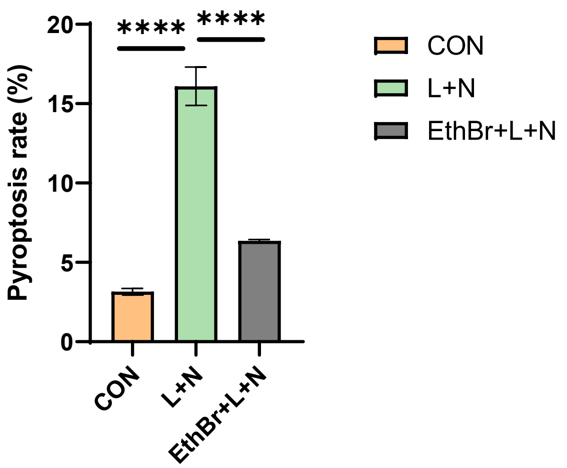

Fig. S13

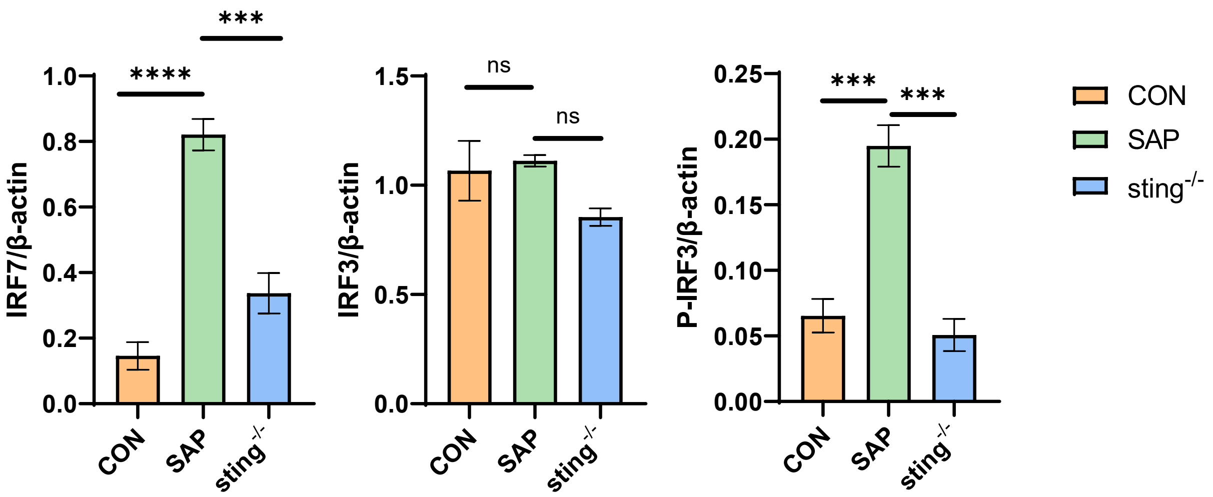

Fig. S15

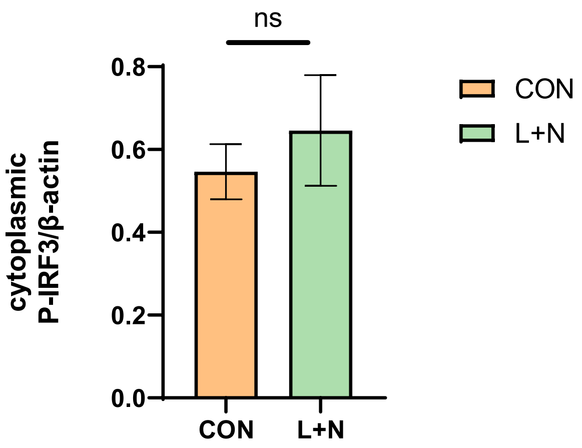

Fig. S14

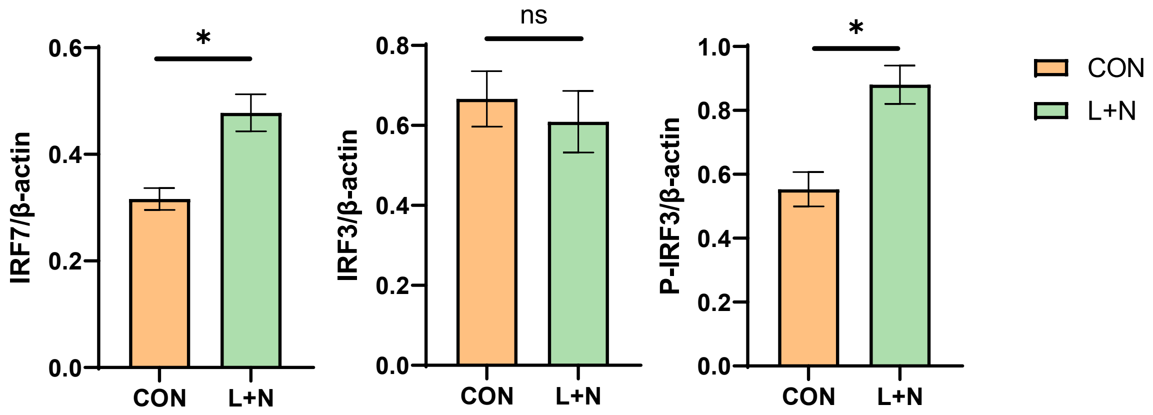

Fig. S16

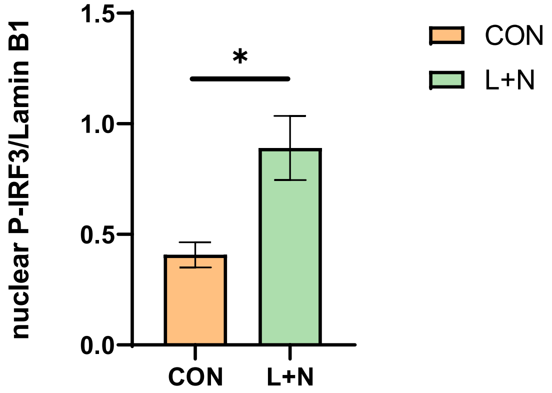

Fig. S17

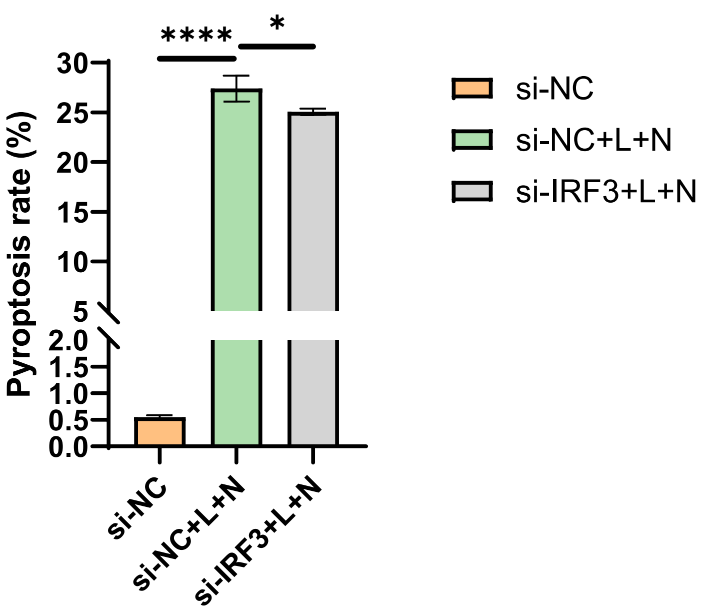

Fig. S18

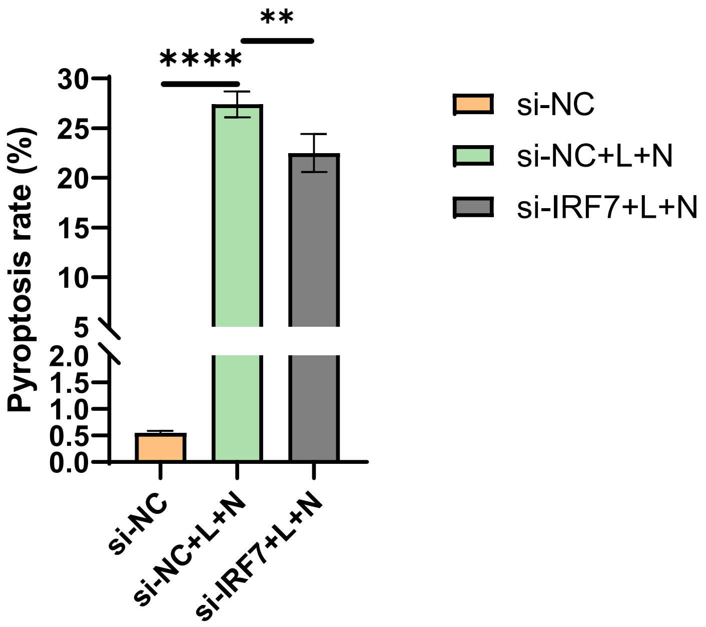

Fig. S19

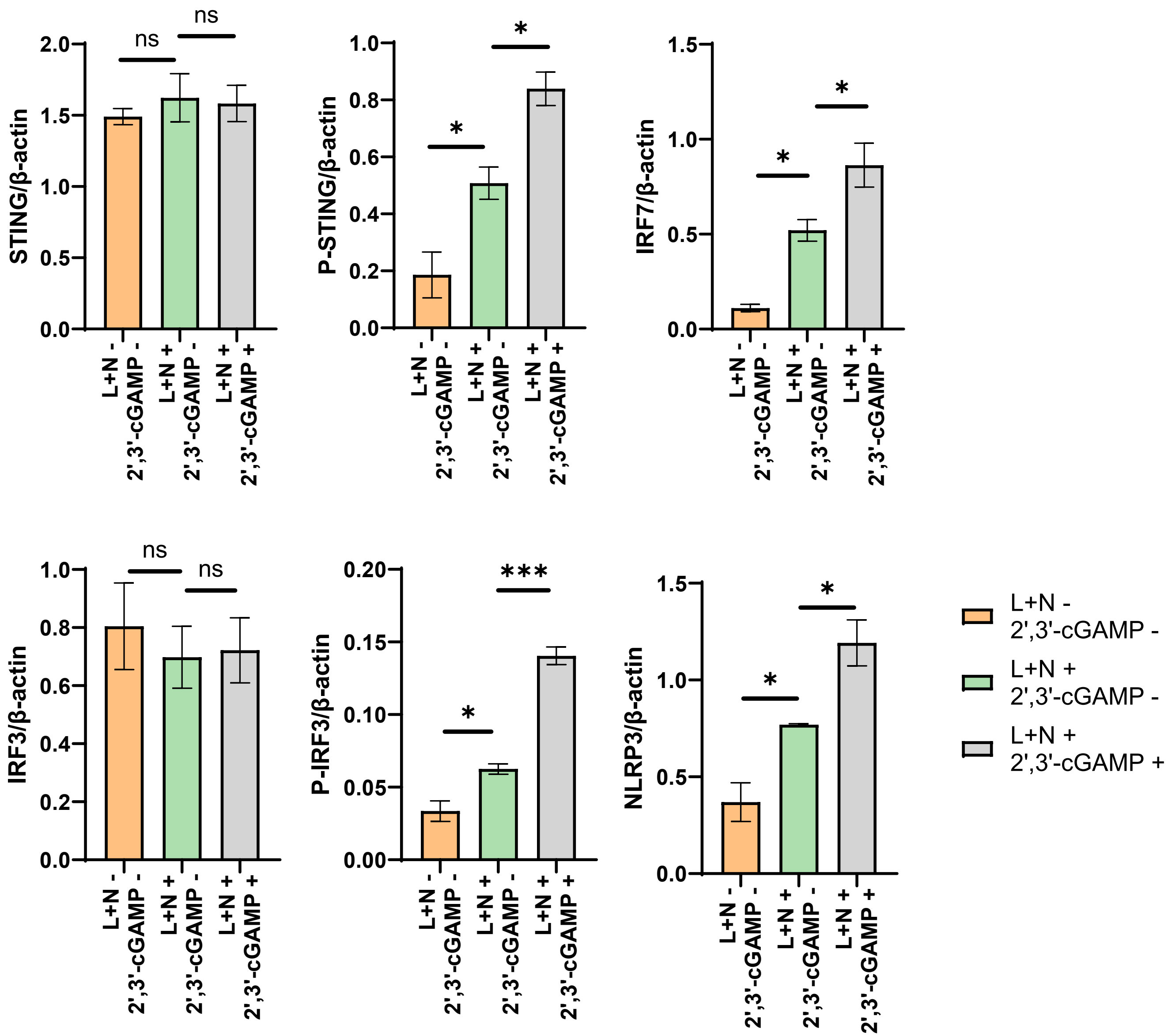
STING/β-actin
P-STING/β-actin
IRF7/β-actin
IRF3/β-actin
P-IRF3/β-actin
NLRP3/β-actin
ns
*
***
L+N -
2',3'-cGAMP -
L+N +
2',3'-cGAMP -
L+N +
2',3'-cGAMP +

Fig. S20

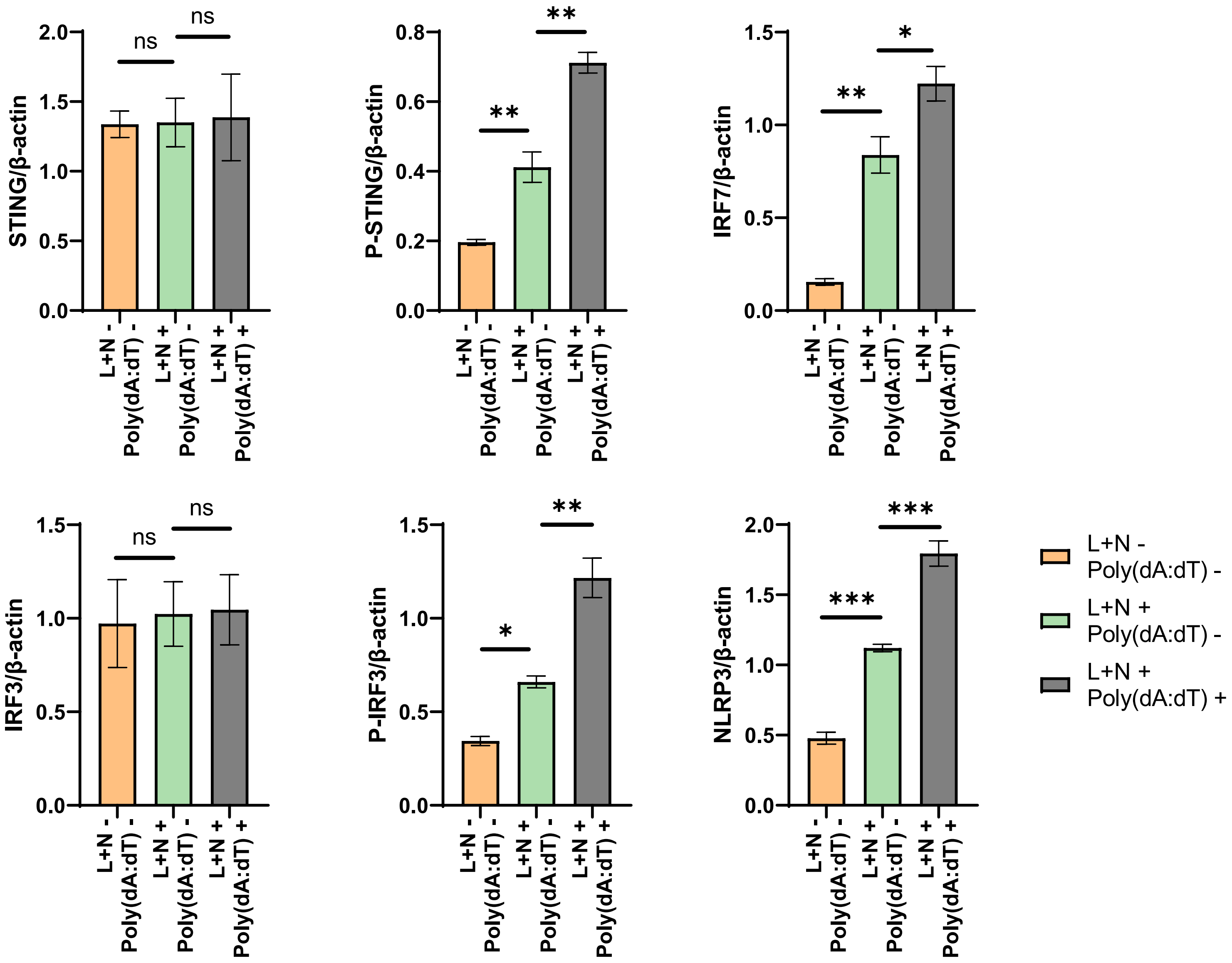
STING/β-actin
ns
ns
P-STING/β-actin
**
**
IRF7/β-actin
**
*
IRF3/β-actin
ns
ns
P-IRF3/β-actin
*
**
NLRP3/β-actin
***
***
L+N -
Poly(dA:dT) -
L+N +
Poly(dA:dT) -
L+N +
Poly(dA:dT) +

Fig. S21

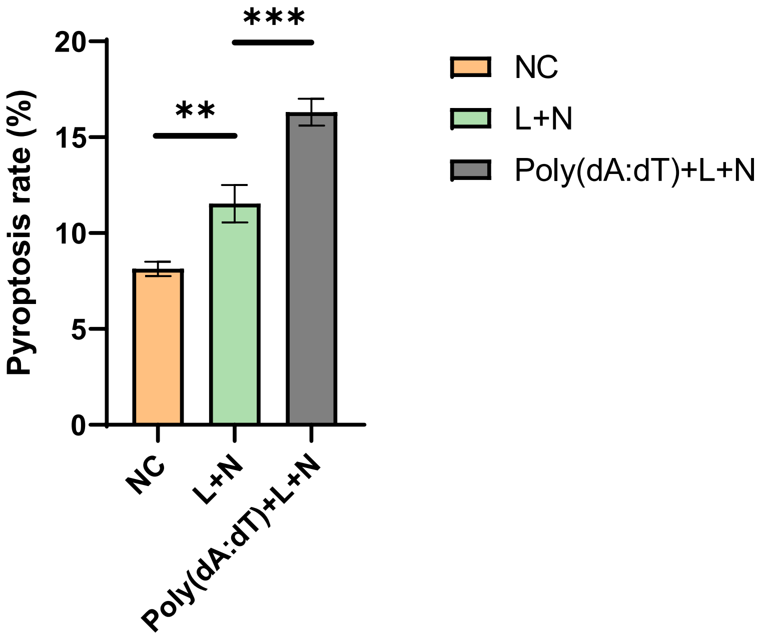

Fig. S22

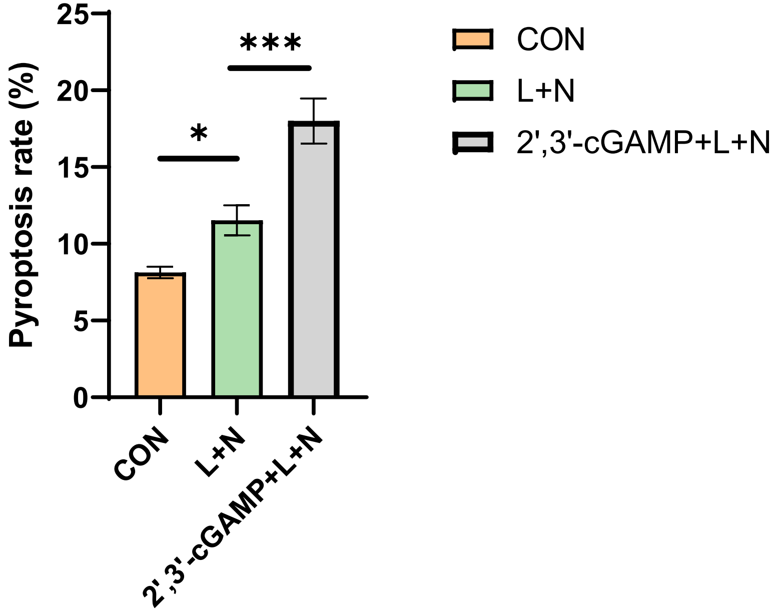

Fig. S23

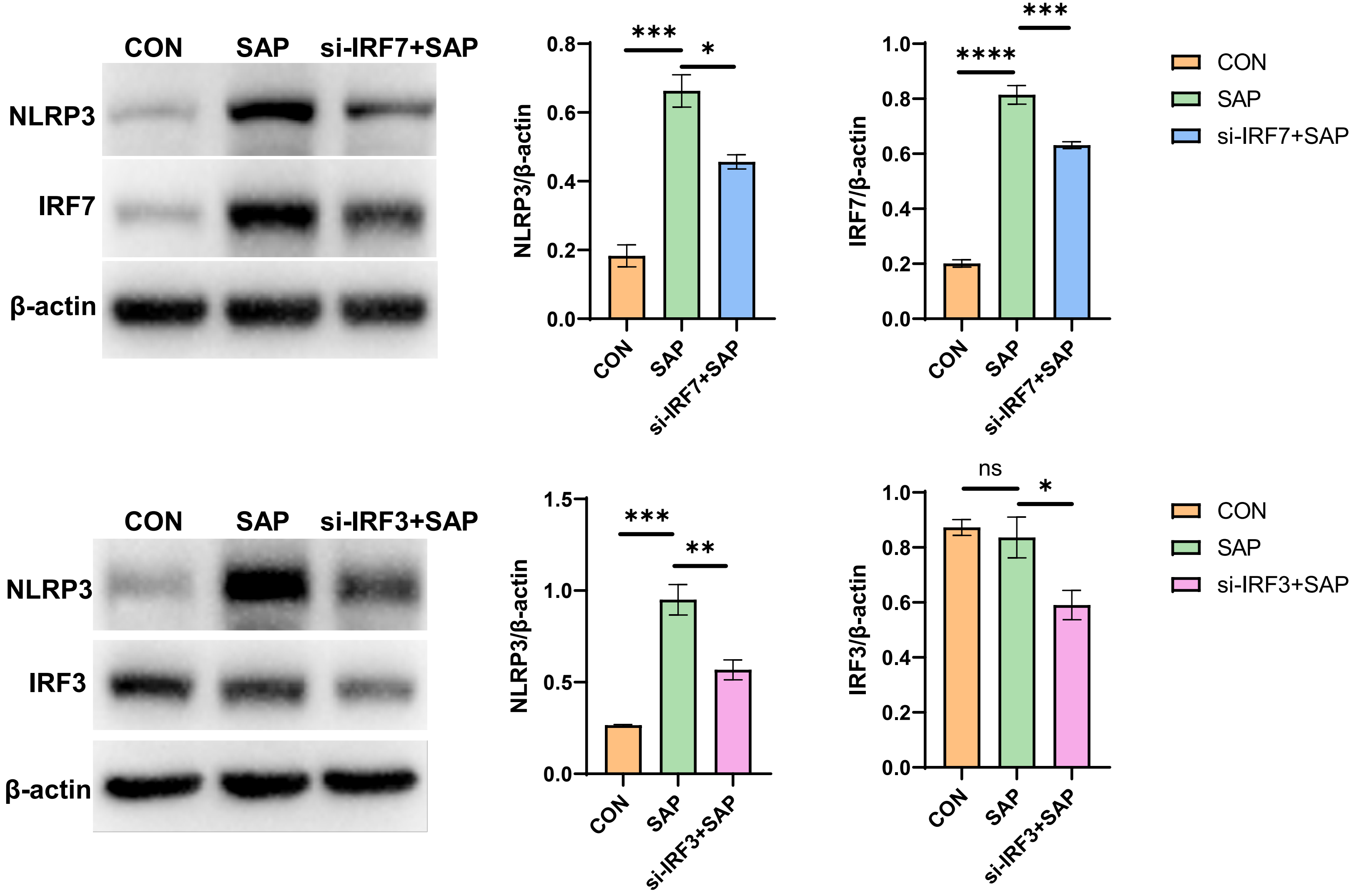

CON
SAP
si-IRF7+SAP
NLRP3
IRF7
β-actin
NLRP3/β-actin
0.0
0.2
0.4
0.6
0.8
***
*
IRF7/β-actin
0.0
0.2
0.4
0.6
0.8
1.0
****
***
CON
SAP
si-IRF7+SAP
si-IRF3+SAP
NLRP3
IRF3
β-actin
NLRP3/β-actin
0.0
0.5
1.0
1.5
***
**
IRF3/β-actin
0.0
0.2
0.4
0.6
0.8
1.0
ns
*
CON
SAP
si-IRF3+SAP

Fig. S24

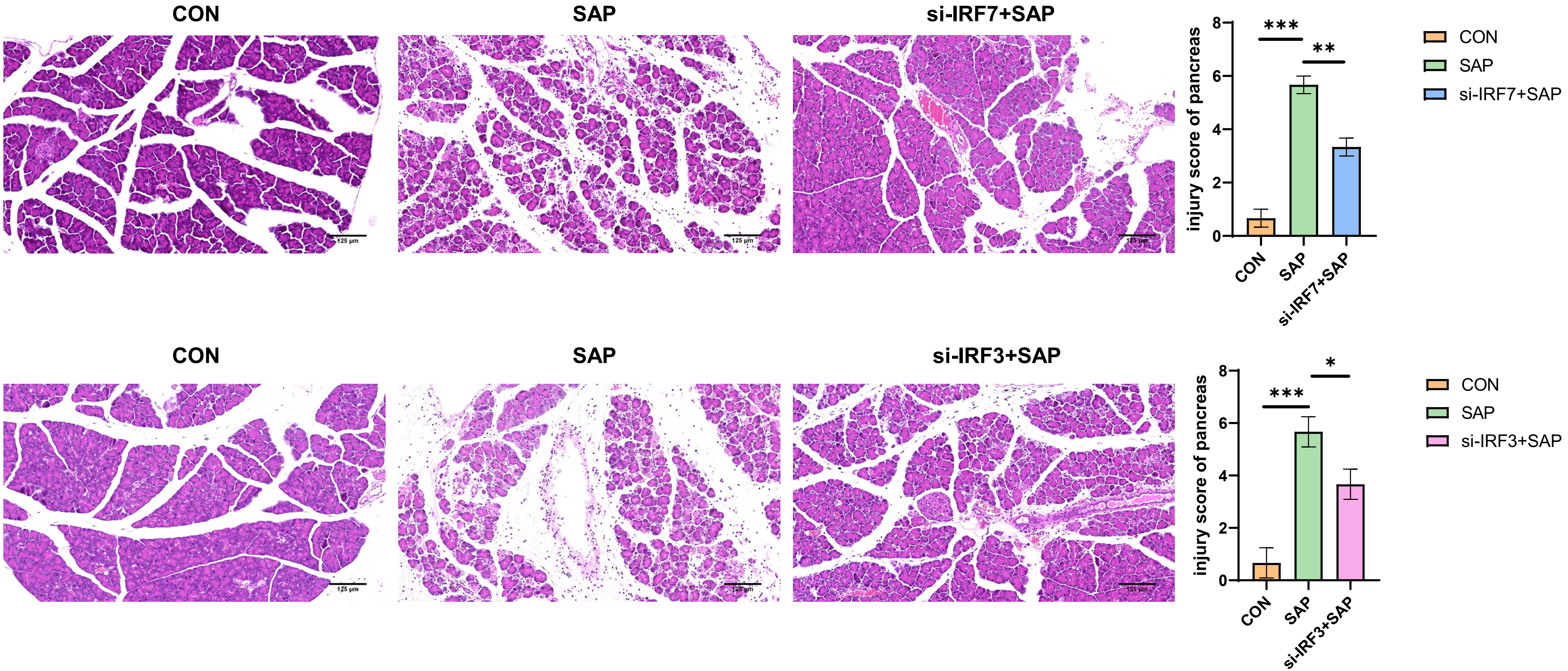

CON
SAP
si-IRF7+SAP
injury score of pancreas
***
**
CON
SAP
si-IRF7+SAP
CON
SAP
si-IRF3+SAP
injury score of pancreas
***
*
CON
SAP
si-IRF3+SAP

Fig. S25

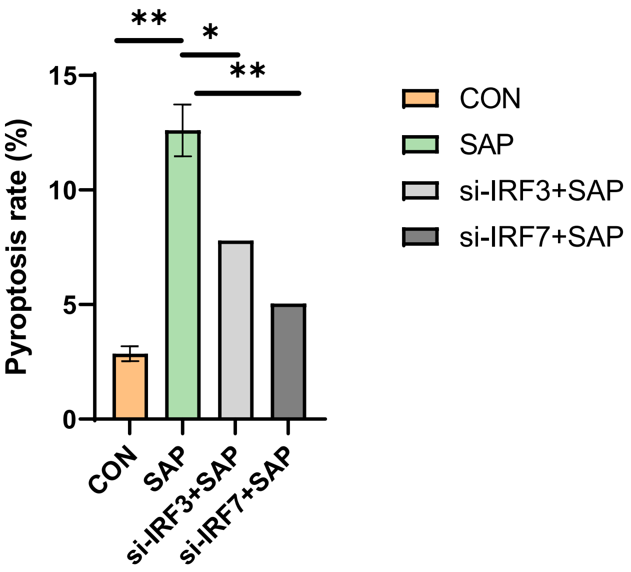

**
*
**
15
10
5
0
Pyroptosis rate (%)
CON
SAP
si-IRF3+SAP
si-IRF7+SAP
CON
SAP
si-IRF3+SAP
si-IRF7+SAP

Fig. S26

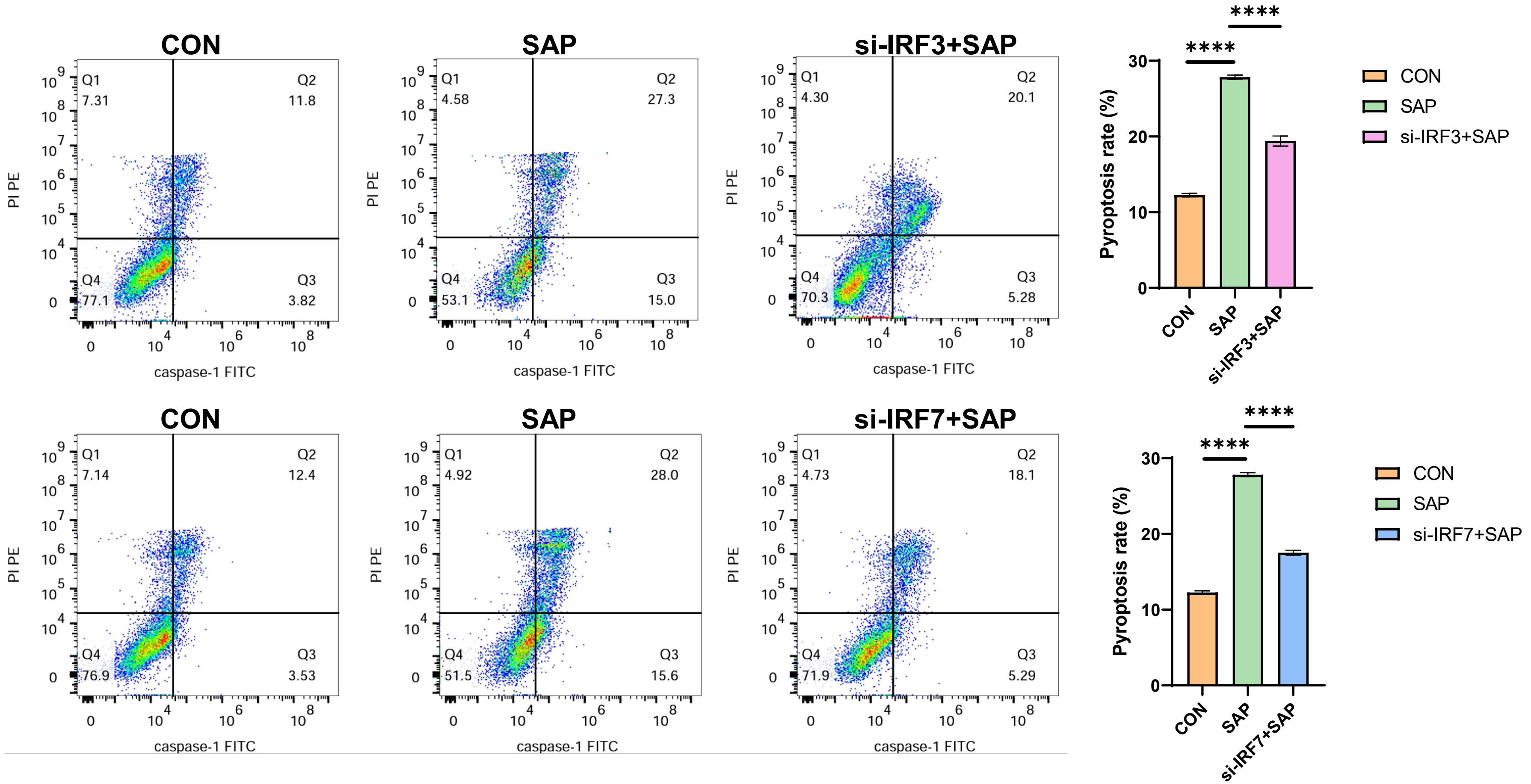
CON
SAP
si-IRF3+SAP
si-IRF7+SAP
Q1
Q2
Q3
Q4
7.31
11.8
77.1
3.82
4.58
27.3
53.1
15.0
4.30
20.1
70.3
5.28
7.14
12.4
76.9
3.53
4.92
28.0
51.5
15.6
4.73
18.1
71.9
5.29
PI PE
caspase-1 FITC
Pyroptosis rate (%)
****
